# *Acer tegmentosum* Maxim and *Bacillus subtilis*-fermented products inhibit TNF-α-induced endothelial inflammation and vascular dysfunction of the retina: the role of tyrosol moiety in active compounds targeting Glu^230^ in SIRT1

**DOI:** 10.3389/fphar.2024.1392179

**Published:** 2024-11-20

**Authors:** Phuc Anh Nguyen, Jong Soon Won, Min Kyung Cho

**Affiliations:** Department of Pharmacology, College of Oriental Medicine, Dongguk University, Gyeongju, Republic of Korea

**Keywords:** *Acer tegmentosum* Maxim, anti-inflammation, NF-κB, SIRT1, tyrosol

## Abstract

*Acer tegmentosum* Maxim (AT) is a medicinal plant used to treat hepatic, neurological diseases, and cancer. However, the beneficial effects of AT on endothelial dysfunction have not been reported yet. In this study, we evaluated the effects of AT and the main compounds against TNF-α-mediated inflammatory responses and their possible mechanism of action. The anti-inflammatory effect and its molecular mechanism were analyzed by adhesion assay, immunoblotting, promoter-luciferase assay, ELISA, RT-PCR, immunocytochemistry, immunoprecipitation, siRNA gene knockdown, docking, and molecular dynamics simulation. AT and its compounds salidroside and tyrosol reduced TNF-α-induced adhesion between monocytes and endothelial cells. Fermentation of AT with *Bacillus subtilis* converted salidroside to tyrosol, which is salidroside’s aglycone. The fermented AT product (ATF) potently inhibited TNF-α-mediated monocyte adhesion with higher potency than AT. AT or ATF abrogated TNF-α-induced expression of adhesion molecules (VCAM-1 and ICAM-1) and production of MCP-1 with the inhibition of phosphorylated MAP kinases. TNF-α-mediated NF-κB transactivation and RelA/p65 acetylation were suppressed by AT and ATF through the interaction of NF-κB with sirtuin-1 (SIRT1), an NAD^+^-dependent histone deacetylase. *Sirt1* gene knockdown diminished the protective effects of AT and ATF against TNF-α-mediated signaling and inflammatory response. Interestingly, SIRT1 protein expression was significantly increased by ATF and tyrosol rather than by AT and salidroside, respectively. Molecular docking showed that the tyrosol moiety is critical for the interaction with Glu^230^ of SIRT1 (PDB ID: 4ZZH and 4ZZJ) for the deacetylase activity. Molecular dynamics revealed that tyrosol can induce the movement of the N-terminal domain toward the catalytic domain of SIRT1. This study demonstrates the potential of AT and ATF to prevent endothelial inflammation and vascular dysfunction of the retina by the MAPK/NF-κB/SIRT1 signaling pathways and targeting of the tyrosol moiety to Glu^230^ in SIRT1.

## 1 Introduction

*Acer tegmentosum* Maxim (Aceraceae) has been used as a medicinal plant to treat liver diseases (such as hepatitis, cirrhosis, and hepatocellular carcinoma) in East Asian countries (Hwang et al., 2013; Chang et al., 2017; Bae et al., 2022). Recently, the Ministry of Food and Drug Safety (MFDS) of South Korea approved AT as a food material without acute toxicity (approval #2018-7), and LD_50_ was reported to be over 2,000 mg/kg in rats (Hwang et al., 2013; Park et al., 2019). *Acer tegmentosum* Maxim extracts (AT) abrogated high-fat diet-induced triglyceride and cholesterol accumulation in animal study (Cho et al., 2020). AT protected H_2_O_2_-induced damage via MAPK signaling pathways in hepatocytes (Park et al., 2019). Salidroside (SAL), the main compound of AT, reduced the hepatic triglyceride content and increased insulin sensitivity in the liver tissues of obese mice or leptin-deficient ob/ob mice (Wang et al., 2016a; Zheng et al., 2018). Tyrosol (TYR), which is SAL’s aglycone, alleviated ROS production in palmitic acid-induced oxidative stress in hepatocytes and reversed dysregulated lipid deposition in high-fat diet-fed mice (Sarna et al., 2016). TYR attenuated the inflammatory response and maintained the capillary barrier (Kim et al., 2017). In addition, TYR could penetrate and accumulate in cells and improve the intracellular antioxidant defense system (Di Benedetto et al., 2007).

Flavonoids and phenolic compounds are large-class secondary metabolites found in plants (Huynh et al., 2014). There are several methods to extract phenolic compounds from plants, including physicochemical and chemical processes. On the other hand, fermentation promotes the release and metabolism of polyphenols from plant sources, which improve their functionality (Huynh et al., 2014). Previously, we reported that *Bacillus subtilis*-fermented products were biotransformed into secondary metabolites, thereby enhancing the antioxidant effect (Gum et al., 2017). The content of SAL in AT extracts is approximately 6%–8%, while the content of TYR is very low (Hwang et al., 2013; Park et al., 2019). *Bacillus subtilis* produces β-glucosidase, which can cause deglycosylation of the glycol residue of SAL, generating its aglycone TYR (Nemeth et al., 2003; Huynh et al., 2014; Gum et al., 2017). *Bacillus subtilis* has been used in the fermentation of natto (Japanese fermented soybeans) and other soybean products to enhance their antioxidant effects (Huynh et al., 2014).

The vascular system plays a critical role between tissue and circulation. Endothelial cells, a major component of the innermost layer of blood vessels, act as a barrier to maintain vascular homeostasis and the circulatory system (Piao et al., 2018). Vascular inflammation is characterized by the activated endothelial cells, which is associated with various diseases, including the cardiovascular complication linked to metabolic syndrome (Lenin et al., 2018; Nguyen et al., 2019). Activated endothelial cells upregulate adhesion molecules such as VCAM-1 and ICAM-1, promoting the recruitment of immune cells to the vascular wall, which exacerbates inflammation and contributes to disease progression (Ouyang et al., 2024). Therefore, therapeutic strategies that effectively target activated endothelial cells are required to reduce vascular inflammation.

Tumor necrosis factor (TNF)-α, an inflammatory cytokine, plays a pivotal role in the disruption of vascular circulation, causing endothelial dysfunction in many pathophysiological conditions including diabetes and metabolic syndrome (Ouyang et al., 2024). TNF-α activates three major MAPK cascades and NF-κB (a transcription factor), mediating the transcription of inflammatory genes (Choi et al., 2018). Under TNF-α stimulation, NF-κB translocates to the nucleus, where it undergoes posttranslational modifications, including phosphorylation, methylation, ubiquitination, and acetylation (Yang et al., 2010). The acetylation at Lys^310^ of RelA/p65 is crucial as it prolongs the stability of RelA/p65 and enhances the full transcriptional activity of NF-κB (Huang et al., 2010). Sirtuin-1 (SIRT1, an NAD^+^-dependent histone deacetylase) deacetylates histone protein such as NF-κB (Chen et al., 2002). Therefore, SIRT1 activation abrogates TNF-α-stimulated endothelial cell inflammation via NF-κB transactivation (Pan et al., 2016). We also previously reported the beneficial role of SIRT1 as a transcriptional regulator of NF-κB in endothelial cells in the anti-inflammatory response (Nguyen et al., 2019).

Since SIRT1 is linked to multiple biological processes, it has been studied as a therapeutic target for metabolic disorders and inflammation (Dai et al., 2015). The first generation of small-molecule sirtuin-activating compounds (STAC) was a group of related plant polyphenols, such as resveratrol and quercetin (Hubbard and Sinclair, 2014). The Glu^230^ residue of the N-terminal domain (NTD) in hSIRT1 plays a critical role in the direct allosteric activation and stabilization of SIRT1 by STAC (Dai et al., 2015). Therefore, compound targeting the Glu^230^ residue of SIRT1 is a good approach to study when studying SIRT1 activation (Hubbard et al., 2013; Dai et al., 2015).

Our present study is the first to evaluate the protective effect of AT against endothelial inflammatory responses by TNF-α and the enhanced anti-inflammatory effect of *B. subtilis*-fermented ATF. Additionally, the role of NF-κB and SIRT1 as a mechanism in the anti-inflammatory effect was identified, and it was proven that the main compounds of the candidates target the Glu^230^ residue of SIRT1 using molecular docking.

## 2 Materials and methods

### 2.1 Reagents

The dried twig of AT was purchased from Omniherb (Gyeongbok, Korea). TNF-α (T6674), anti-β-actin antibody (A1978), thiazolyl blue tetrazolium bromide (MTT) (M2128), streptozotocin (S0130), poly-L-lysine (P4707), and bovine serum albumin (BSA) (A8806) were purchased from Sigma-Aldrich (St. Louis, MO, United States). Lipofectamine 2000, goat anti-rabbit IgG (#65-6120), and goat anti-mouse IgG (#62-6520) were purchased from Invitrogen (Carlsbad, CA, United States). Anti-intercellular adhesion molecule-1 (ICAM-1) (sc-7891 and sc-8439), anti-NF-κB (sc-8008), anti-SIRT1 (sc-74504), anti-vascular cell adhesion molecule-1 (VCAM-1) (sc-8304) antibodies, anti-IgG-FITC (sc-2010), protein G agarose (sc-2002), DAPI (sc-3598), and Immuno *In situ* Mount (sc-45088) were obtained from Santa Cruz Biotechnology (CA, United States). Calcein-AM (C3100MP), goat anti-mouse Alexa Fluor 488 (A32723), and goat anti-rabbit Alexa Fluor 568 (A11011) were purchased from Thermo Fisher Scientific (Waltham, MA, United States). Anti-phospho-ERK1/2 (#9101), anti-ERK1/2 (#9102), anti-phospho-p38 MAPK (#9211), anti-p38 MAPK (#9212), anti-phospho-SAPK/JNK (#9255), and anti-JNK2 (#9258) antibodies were obtained from Cell Signaling Technology (Beverly, MA, United States). All materials used for SDS-PAGE were purchased from Bio-Rad (Hercules, CA, United States). The *Sirt1* small interfering RNA (siRNA, sc-40986) and non-targeting scrambled RNA (control siRNA, sc-37007) were obtained from Santa Cruz Biotechnology (Sparks, MD, United States).

### 2.2 AT extraction and fermentation

Extraction of AT was performed as previously reported, with minor modifications (Yu et al., 2010). AT (400 g) was powdered and extracted with 2.5 L of 70% ethanol at 80°C for 2 h. The ethanol extract was filtered through a Whatman #2 filter paper (Whatman International, Maidstone, United Kingdom) and concentrated using a rotary evaporator (model VV2000; Heidolph, Walpersdorfer, Germany) to obtain the final extraction. The samples were frozen in −70°C before being freeze-dried using a FD8505S freeze-dryer (Ilshin Biobase Co., Ltd., Busan, South Korea), and a voucher (AT-0100) specimen was deposited in the pharmacology laboratory at the College of Oriental Medicine, Kyungju, South Korea. The final yield of the ethanol extract was 19.6%. The content of salidroside, the major component of AT, was calculated from the relevant peak area using an external standard method and quantified as 6.41% (w/w) in AT (Bae et al., 2022) (Supplementary Figure 2).

A total of 300 mg AT extract was fermented with 100 mL Luria–Bertani (LB) media containing 0.2–20% *B. subtilis* NCDO 1769T (X60,646) culture for 7 days (ATF) and then frozen at −70°C before use (ATF-0100) (Gum et al., 2017). Negative AT samples were subjected to 100 mL LB media without *B. subtilis* for 7 days (AT). After fermentation, both AT or ATF samples were stored in −70°C for further experiments.

### 2.3 HPLC analysis

HPLC analyses were performed as previously published with minor modifications (Meng et al., 2006). The HPLC analyses of samples were carried out using an Agilent 1260 HPLC system (1260 Infinity II; Agilent Technologies, Santa Clara, CA, United States) coupled to the Agilent G7115A-1260 DAD WR detector (diode array detector). In brief, 10 μL of AT or ATF samples (3 mg/mL) were injected into the Agilent ZORBAX Eclipse plus C18 column (4.6 mm × 250 mm, 5 μm) at 25°C. The mobile phase was 0.1% formic acid in H_2_0 (A) and 100% MeOH (B), run at 1 mL/min; peaks were detected with a UV detector at 225/278 nm. The ratios of the A/B solvent were 80/20, 80/20 to 0/100 (linear gradient), and 0/100 at running times of 0–10, 11–30, and 31–35 min, respectively. SAL or TYR at 500 ppm was used as the standard. The SAL and TYR peaks were observed at 7.5 and 9.5 min, respectively.

### 2.4 Cell culture

EA. Hy926, a human umbilical vein endothelial cell line fused with the human carcinoma cell line (CRL-2922), was purchased from the ATCC (Rockville, MD, United States). Cells were maintained in Dulbecco’s modified Eagle’s medium (DMEM) (LM 001-05, Welgene, Gyeongsangbuk-do, Korea) containing 10% FBS, 50 μg/mL streptomycin, and 50 units/mL penicillin at 37°C in a humidified 5% CO_2_ atmosphere. THP-1 cells (ATCC^®^ TIB-202™), a monocyte cell line, were purchased from the ATCC (Rockville, MD, United States). The cells were cultured in RPMI 1640 medium (LM 011-01, Welgene, Gyeongsangbuk-do, Korea) containing 10% FBS, 0.05 mM 2-mercaptoethanol, 50 μg/mL streptomycin, and 50 units/mL penicillin. Cells were treated with TNF-α for the indicated time with or without candidates (AT, ATF, SAL, or TYR) pretreated for 1 h.

### 2.5 Cell viability assay

Cells were seeded in a 96-well culture plate. After reaching 80% of confluence, cells were incubated with candidates at different concentrations for 24 h. The MTT solution (5 mg/mL) was incubated for 2 h. The assays were stopped by adding DMSO, and the cell viability was observed at 520 nm using a Sunrise™ Absorbance microplate reader (Tecan, Bionics, Seoul, Korea).

### 2.6 Adhesion assay

The monocyte–endothelial interaction was evaluated by the measurement of fluorescent labeling monocytes, as previously described (Nguyen et al., 2019). Endothelial cells were seeded in a 96-well culture black plate and then incubated with candidates for 1 h, following exposure of 10 ng/mL TNF-α for 6 h. Human monocyte cells were labeled with calcein-AM at 37°C for 1 h. Calcein-AM-labeled cells were co-cultured with the monolayer of endothelial cells for 1 h. After the incubation, the recruited monocytes were measured using microplate reader (Bio-Tek Instruments, Winooski, VT, United States) with excitation wavelength 490 nm and emission wavelength 510–570 nm. The average fluorescence intensity represented the number of monocytes adhered to endothelial cells.

### 2.7 Enzyme-linked immunosorbent assay (ELISA)

MCP-1 cytokine levels in the culture supernatants were detected by ELISA using a commercial Human MCP-1 ELISA kit (Pierce Biotechnology, IL, United States), according to manufacturer’s protocol. The OD values were measured using a Sunrise™ Absorbance microplate reader (Tecan, Bionics Co., Seoul, Korea). Concentrations of MCP-1 cytokines were extrapolated from the standard curves provided.

### 2.8 RT-PCR

RT-PCR was performed as previously reported (Kim et al., 2008). The primers encoding MCP-1 (forward primer 5′ -GAT​GCA​ATC​AAT​GCC​CCA​GTC -3′ and reverse primer 5′- TTT​GCT​TGT​CCA​GGT​GGT​CCA​T -3′) and β-actin (forward primer 5′- GACTACCTCATGAAGATC -3′ and reverse primer 5′- GATCCACATCTGCTGGAA -3′) were synthesized from Bioneer (Cheongwon, Korea).

### 2.9 Immunoprecipitation and immunoblotting

Immunoprecipitation and immunoblotting were performed, as previously reported (Nguyen et al., 2019). For immunoprecipitation, cell lysates were incubated with antibodies against SIRT1 or NF-κB at 4°C for 2.5 h and protein G plus-agarose beads at 4°C for 1 h. The immunocomplexes were washed with IP buffer and dissolved by boiling. Samples were then run on SDS-PAGE gels. In immunoblotting, primary antibodies were incubated at 4°C overnight, and secondary antibodies were incubated at room temperature for 1 h. The target proteins were developed using an ECL chemiluminescence detection kit (Amersham Biosciences, Bucks, United Kingdom). The protein expressions were normalized with β-actin immunoblot. The densitometry was measured using Gel-Pro Analyzer software (Media Cybernetics, MD, United States).

### 2.10 Promoter-luciferase assay

The full-length VCAM-1 and ICAM-1 firefly luciferase reporter gene construct (VCAM-1: -1,716 to +119 bp, ICAM-1: -1,350 to +45 bp) were transfected as previously reported (Kim et al., 2008; Nguyen et al., 2019). The luciferase activities in cell lysates were measured using a GloMax 20/20 Luminometer (Tuner BioSystems, Sunnyvale, United States). The relative luciferase signal was normalized by dual transfection with the pSV-β-galactosidase control vector.

### 2.11 Immunocytochemistry

Cells were seeded in an 8-well chamber slide system (Nunc Lab-Tek II, Seoul, Korea). Immunofluorescence-labeled cells were evaluated as previously reported (Wang et al., 2016b). After drug treatment for the indicated time, cells were fixed with ice-cold MeOH for 15 min. After permeabilizing and blocking, the anti-NF-κB primary antibody was incubated overnight at 4°C. The secondary antibody against anti-mouse-FITC was then incubated. The images were taken in random regions using a BioTek Lionheart FX Automated Microscope (Agilent, VT, United States).

### 2.12 Transfection of siRNAs

Cells were transfected with siRNAs using Lipofectamine 2000 (Invitrogen, Carlsbad, CA, United States), following the manufacturer’s protocol. Human *Sirt1* or scrambled siRNA (10 nM) was transiently transfected for 3 h to knock down *Sirt1*. After an 18 h-recovery period, promoter-luciferase assay, immunoblotting, and adhesion assays were performed, as described above.

### 2.13 Protein sequence alignment

The full sequence alignment of human SIRT1 (NP_036370.2) was obtained from NCBI by FASTA. The crystal structures of SIRT1 PDB IDs 4ZZH and 4ZZJ were retrieved from the Protein Data Bank online database (PDB). The sequences were imported, and the structure-based sequence alignment was performed using BioEdit software version 7.2 (Pannek et al., 2017).

### 2.14 Molecular docking

Three-dimensional sequences of SIRT1 with the NTD region were retrieved from PDB: 4ZZH and 4ZZJ (www.rcsb.org). All structures were optimized by the removal of ligands, substrates, ions, and water molecules. The active compounds’ (SAL and TYR) structures were downloaded from the NCBI PubChem online database (http://pubchem.ncbi.nlm.nih.gov/) and energy minimized. Molecular docking of PDB and compounds was analyzed by AutoDockZn and PyRx softwares, to predict the binding affinity and ligand conformational change. The proper docking conformations were chosen based on binding affinity scores and the root-mean-square deviation (RMSD) values. The binding pockets and interactions between the ligand and protein were visualized by PyMOL and Biovia Discovery Studio 2021 software applications.

### 2.15 Molecular dynamics simulation

The docked complexes of the active compounds and SIRT1 were used as the initial coordinates for molecular dynamics (MD) simulations using the GROningen MAchine for Chemical Simulations (GROMACS) (Shamsi et al., 2024). MD simulations were performed with GROMACS version 5.1.2 with the CHARMM36-jul2022 force field. The complexes were immersed in a dodecahedron box and solvated using the CHARMM-modified TIP3P water model. The topologies of SAL and TYR were generated using the CHARMM General Force Field (CGenFF). The systems were neutralized with counter ions. The energy minimization process was initiated with restraints on both the ligand and protein, followed by an unrestrained minimization step using the steepest descent method. The equilibration phases were conducted by NVT ensemble for 100 ps, followed by NPT ensemble for an additional 100 ps, with the temperature maintained at 300 K. The final MD trajectories were run for 100,000 ps (100 ns) with a 2.0-fs time step. Post-simulation analysis was performed using the following GROMACS utilities: root-mean-square deviation (RMSD), root-mean-square fluctuation (RMSF), radius of gyration (Rg), solvent-accessible surface area (SASA), and hydrogen bond analysis. The conformational changes in the ligand–protein complexes were visualized at the 20-ns interval using PyMOL software.

### 2.16 Mouse experiments

The animal study was conducted in accordance with the institutional guidelines based on the principles of laboratory animal care (NIH publications No. 85-23, revised 1996) and Korean laws of laboratory animal care (Health & Welfare Committee Registration No. 9025, revised 2010) and was approved by the Institutional Animal Care and Use Committee of the Dongguk University (protocol number IACUC-2023-15). Male C57Bl/6JBomTac mice were purchased from Daehan Bio Link (Chungbuk, South Korea). The 5-week-old mice received three consecutive intraperitoneal injections of streptozotocin (STZ) at a dose of 55 mg/kg in citrate buffer (0.1 M, pH 4.5). One week after STZ-treatment, mice with glucose levels exceeding 250 mg/dL were divided into four groups: control, STZ, STZ + AT, and STZ + ATF. AT or ATF (50 mg/kg) was orally administered daily for 4 weeks. All mice were anesthetized with pentobarbital sodium, and their eyes were collected and stored in 4% paraformaldehyde until further processing.

### 2.17 Histological evaluation

Eye samples were soaked in 30% sucrose (0.2 M phosphate buffer) at 4°C overnight before fixing in the optimal cutting temperature (OCT) compound for cryosectioning. Retina radial sections (8 μm) were placed on poly-L-lysine-coated slides and heated at 60°C for 2 h. Samples were permeabilized with washing buffer (0.1% Triton X-100) at room temperature for 20 min and then rinsed with PBS twice for 5 min. Retinal samples were blocked with buffer containing 5% fetal bovine serum, 2% BSA, and 0.1% Triton X-100 at room temperature for 30 min. Tissue samples were stained with primary antibodies for VCAM-1 and ICAM-1 (1:400 dilution) overnight at 4°C. The sections were then incubated with secondary antibodies conjugated to goat anti-mouse Alexa Fluor 488 and goat anti-rabbit Alexa Fluor 568 (1:600 dilution) for 30 min at room temperature. For counterstaining, DAPI was incubated with the sections for 5 min. Sections were analyzed using the BioTek Lionheart FX Automated Microscope (Agilent, VT, United States).

### 2.18 Statistical analysis

Statistical analyses were performed using SPSS 13.0. (IBM SPSS, Chicago, United States), and all data are presented as the mean ± standard error (S.E.). The statistical comparisons between the groups were determined using one-way ANOVA, followed by Dunnett’s *post hoc* test.

## 3 Results

### 3.1 Effects of AT, SAL, and TYR on TNF-α-induced monocyte–endothelial interactions

**FIGURE 1:**
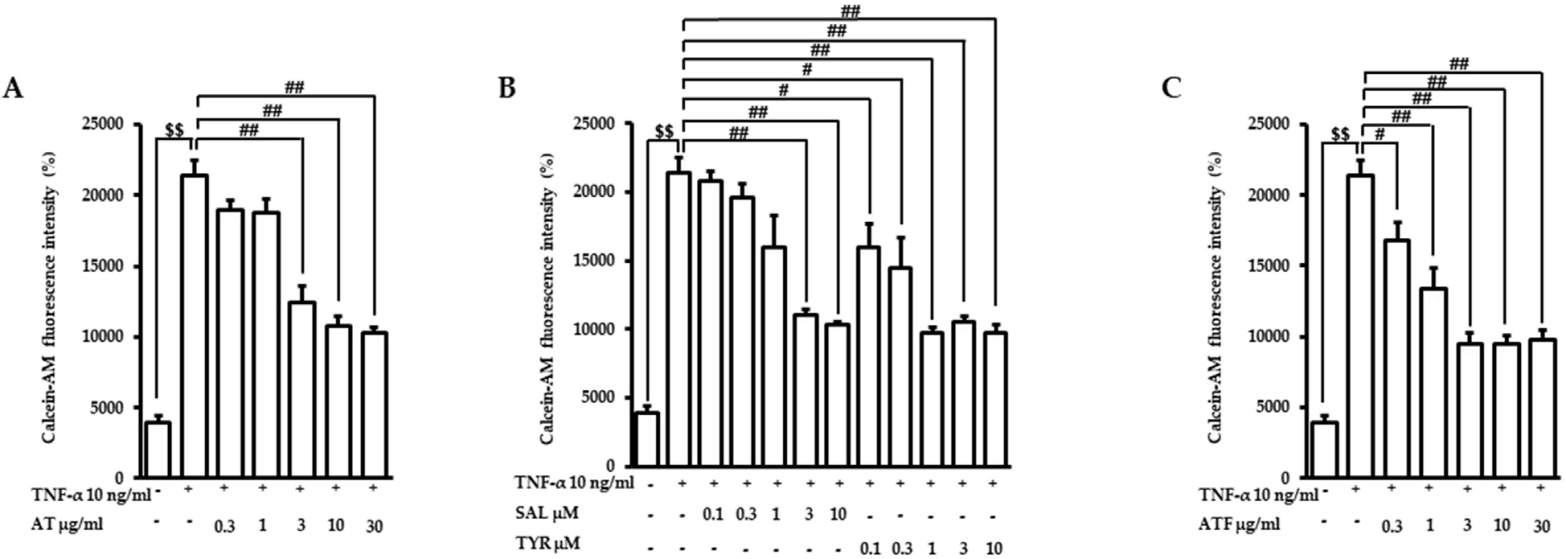
Effects of candidates on TNF-α-induced monocyte recruitment to endothelial cells. Cells were pretreated with **(A)** AT, **(B)** SAL or TYR, and **(C)** ATF at the indicated doses for 1 h and followed by TNF-α incubation for 6 h. Calcein-AM-labeled monocytes were co-cultured with endothelial cells for the next 1 h. The monocyte-attached endothelial cells were evaluated based on fluorescence intensities. (^$$^ *p* < 0.01 for control vs. TNF-α; ^#^ *p* < 0.05, ^##^ *p* < 0.01 for TNF-α vs. the candidate-treated group).

In vascular inflammatory conditions, circulating monocytes bind and migrate through the blood vessels’ endothelial monolayer, which plays a pivotal role in the initiation of vascular dysfunction (Nguyen et al., 2019). Therefore, we investigated the effects of AT, SAL (the main bioactive compound of AT), and TYR (the aglycone of SAL) on the TNF-α-stimulated adhesion of monocytes to endothelial cells. The concentrations of candidates used in this study did not affect cell viability (Supplementary Figure 1). Endothelial cells were pretreated with AT, SAL, or TYR for 1 h, and following TNF-α exposure for the next 6 h. We then measured the attached calcein-AM-labeled monocytes to the endothelial cells. TNF-α induced monocyte adhesion (∼5.5-fold) compared to the control (Figure 1). AT, SAL, and TYR suppressed the elevated monocyte attachment by TNF-α with IC_50_ values of 1.62 μg/mL, 0.94 μM, and 0.14 μM, respectively (Figures 1A, B). TYR exhibited high potency in reducing TNF-α-induced monocyte recruitment on endothelial cells. These results suggest that TYR may exert beneficial effects as an active metabolite in inhibiting monocyte adhesion to endothelial cells.

### 3.2 Effects of AT and ATF on TNF-α-induced adhesion molecule expression and transactivation

SAL is the major compound of AT, whereas TYR is very minor (Hwang et al., 2013; Lee et al., 2014). We previously demonstrated that fermentation with *B. subtilis* subsp. *subtilis* NCDO 1769T (X60646) showed enhanced beneficial effects through secondary metabolite production (Gum et al., 2017). Therefore, we performed AT fermentation with *B. subtilis* subsp. *subtilis* NCDO 1769T (X60646). The fermented product of AT showed a bioconversion from SAL to TYR (Supplementary Figure 2), analyzed by HPLC. In ATF, SAL and TYR contents were 4.44% (w/w) and 0.75% (w/w), respectively. The elevation of monocyte recruitment under TNF-α exposure was inhibited by ATF with an IC_50_ value of 0.49 μg/mL (Figure 1C). The lower IC_50_ value of ATF indicates that ATF is more effective than AT in inhibiting monocyte attachment to endothelial cells, which may be attributed to the biotransformation of SAL in AT.

**FIGURE 2:**
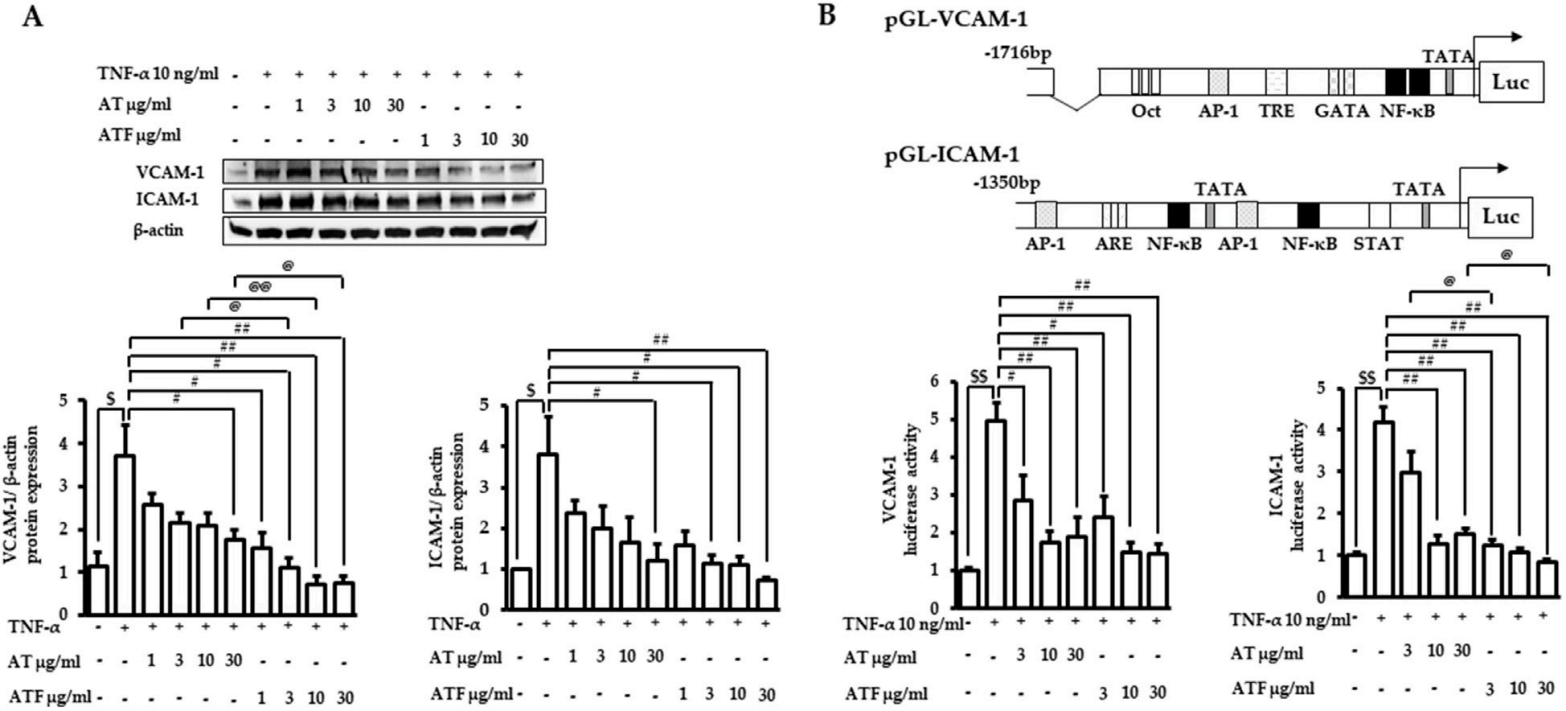
Inhibitory effects of AT and ATF on TNF-α-induced adhesion molecules. **(A)** Cells were pretreated with AT or ATF for 1 h, followed by TNF-α incubation. VCAM-1 or ICAM-1 protein expressions were determined after 12 or 24 h of TNF-α incubation, respectively. Cell lysates were analyzed by immunoblotting using specific antibodies against VCAM-1 and ICAM-1. The protein amounts in each lane were normalized to the β-actin control density. The data were obtained from multiple analyses (n = 4). **(B)** Cells were transfected with VCAM-1 or ICAM-1 promoter constructs. After 18 h of recovery, cells were incubated with AT or ATF for 1 h and then TNF-α for the additional 18 h. Cell lysates were used for the reporter gene assay. The data were obtained from multiple analyses (n = 3). (^$^ *p* < 0.05, ^$$^ *p* < 0.01 for control vs. TNF-α; ^#^ *p* < 0.05, ^##^ *p* < 0.01 for TNF-α vs. candidate-treated group; ^@^ *p* < 0.05, ^@@^ *p* < 0.01 for the same concentration between each candidate).

TNF-α induces endothelial cell activation, leading to transactivation of adhesion molecules such as VCAM-1 and ICAM-1, which represents the initial step in vascular inflammation (Nguyen et al., 2019). The previous report showed that TNF-α increased VCAM-1 expression, which peaked at 12 h, while ICAM-1 expression showed a sustained induction pattern until 24 h (Nguyen et al., 2019). Therefore, we performed immunoblotting with VCAM-1 and ICAM-1 after the indicated times of TNF-α incubation with AT or ATF pretreatment. The protein expressions of VCAM-1 and ICAM-1 were elevated ∼4-folds under TNF-α exposure, which were significantly inhibited by AT or ATF (Figure 2A).

We performed reporter gene assays in cells transfected with the promoter of VCAM-1 or ICAM-1 to determine transcriptional activity under TNF-α exposure. TNF-α increased the luciferase activity of VCAM-1 by 5-fold and ICAM-1 by 4-fold (Figure 2B). Incubation with AT or ATF strongly attenuated TNF-α-mediated transactivation. AT and ATF inhibited VCAM-1 luciferase activity with a similar effective range, with IC_50_ values of 2.86 μg/mL and 2.09 μg/mL, respectively. The stronger protective effect of ATF was shown in ICAM-1 luciferase activity with an IC_50_ value of 1.27 μg/mL compared to AT with an IC_50_ value of 3.09 μg/mL. The significant suppression of ATF in the attenuation of TNF-α-mediated transcriptional activation of adhesion molecules was consistent with the inhibition of those expressions.

### 3.3 Effects of AT and ATF on TNF-α-induced pro-inflammatory cytokine production

**FIGURE 3:**
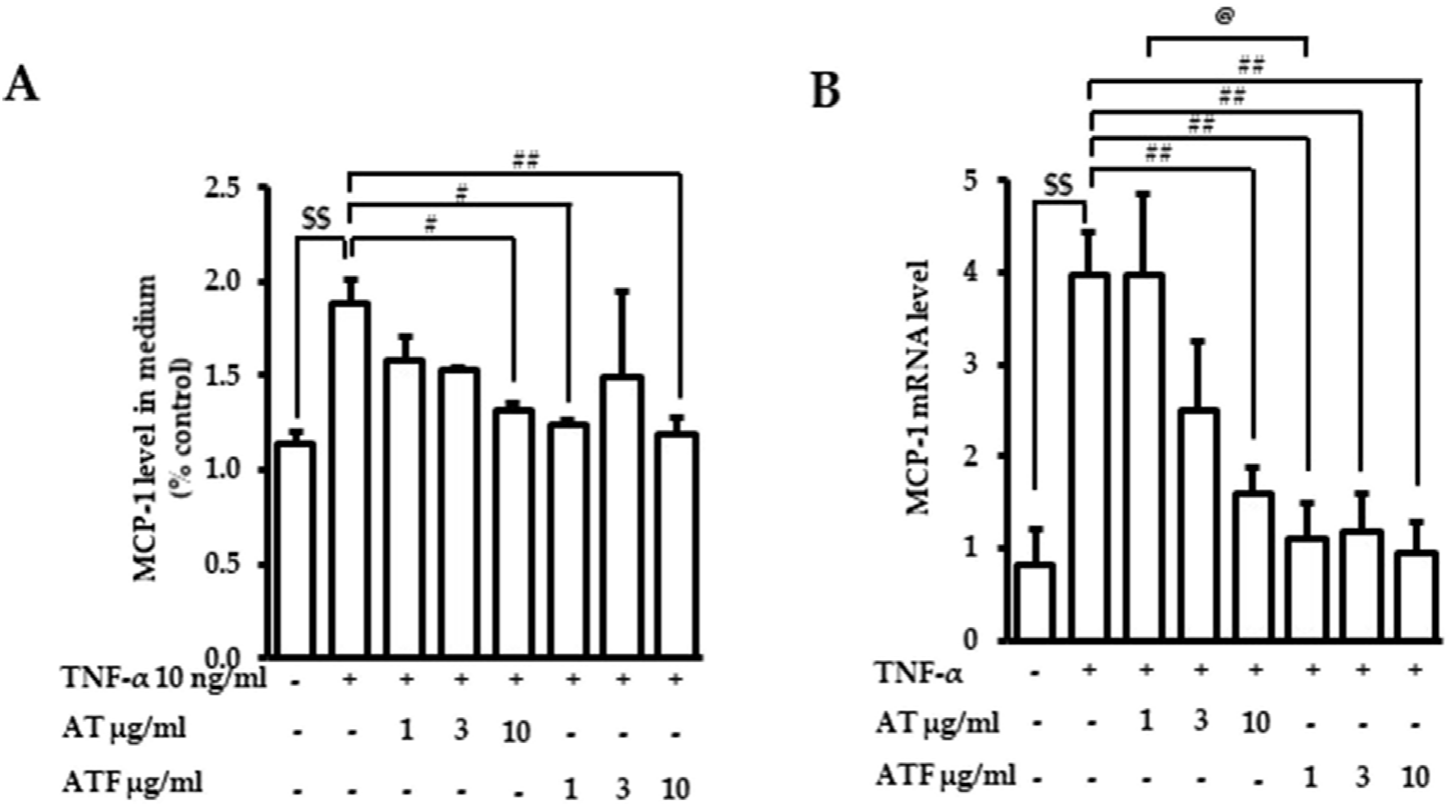
Effects of AT and ATF on TNF-α-induced MCP-1 production and mRNA level. Cells were treated with AT or ATF at the indicated doses for 1 h before TNF-α incubation. **(A)** The MCP-1 level was measured after 36 h of TNF-α treatment with or without AT or ATF by ELISA. The data were obtained from multiple analyses (n = 3). **(B)** The mRNA level of MCP-1 was measured after 6 h of TNF-α exposure by RT-PCR. The data were obtained from multiple analyses (n = 5). (^$$^ *p* < 0.01 for control vs. TNF-α; ^#^ *p* < 0.05, ^##^ *p* < 0.01 for TNF-α vs. candidate-treated group; ^@^ *p* < 0.05 for the same concentration between each candidate).

MCP-1 is one of the key cytokines that regulates the migration and infiltration of monocytes/macrophages, which initiates further inflammatory cellular responses (Tedgui and Mallat, 2006). MCP-1 is induced and secreted in endothelial cells stimulated by TNF-α (Meng et al., 2022). Therefore, we tested whether AT or ATF prevented TNF-α-induced MCP-1. We first measured MCP-1 secretion in the culture media after TNF-α incubation for 36 h. AT or ATF pretreatment abrogated TNF-α-induced MCP-1 production (Figure 3A). Next, we evaluated the level of MCP-1 mRNA. After 6 h of TNF-α treatment, MCP-1 mRNA levels increased 4-fold (Figure 3B). AT and ATF significantly suppressed MCP-1 mRNA, and inhibition by ATF was stronger than that by AT. These results support the hypothesis that ATF is potent due to its conversion to the active compound TYR.

### 3.4 Upstream signaling of the protective effects of AT and ATF against TNF-α exposure

**FIGURE 4:**
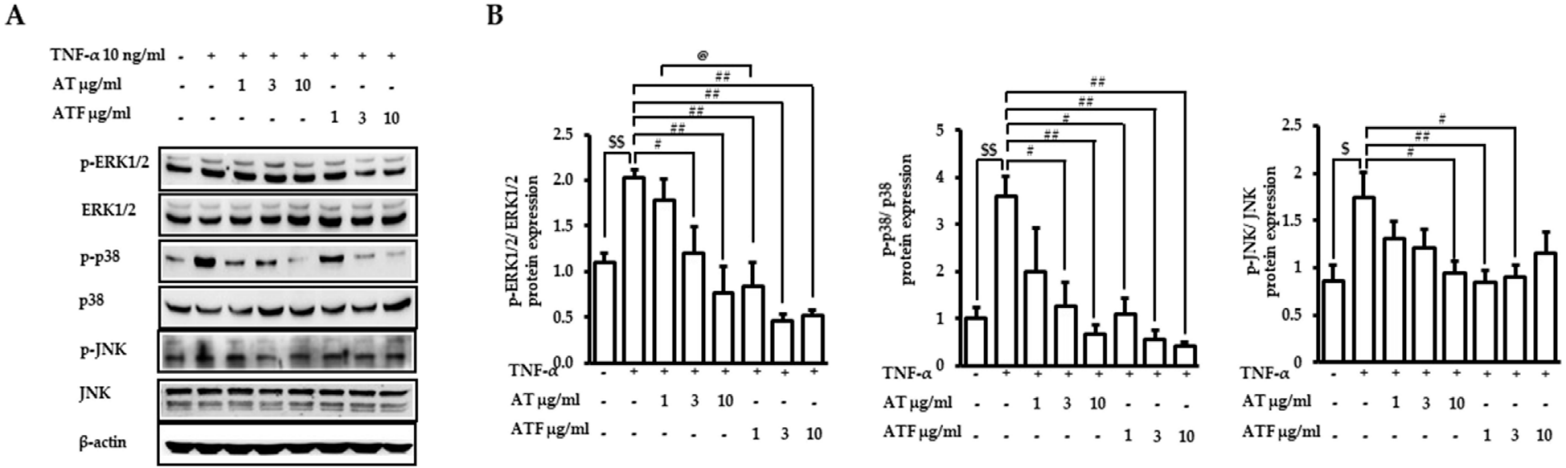
Effects of AT and ATF in the TNF-α-induced MAPK signaling pathway. **(A)** Cells were pretreated with AT or ATF for 1 h and incubated with TNF-α for 30 min or 5 h, as described in the *Materials and methods* section. The expressions of phosphorylated and total MAPKs in cell lysates were detected by Western blot. **(B)** Quantitative analyses of the densitometric intensity of immunoblotting were carried out to quantify MAPK levels. The relative expression ratios of each p-ERK, p-p38 MAPK, and p-JNK were normalized to each EKR, p38 MAPK, and JNK. The data were obtained from multiple analyses (n = 4). (^$^ *p* < 0.05, ^$$^ *p* < 0.01 for control vs. TNF-α; ^#^ *p* < 0.05, ^##^ *p* < 0.01 for TNF-α vs. candidate-treated group; ^@^ *p* < 0.05 for the same concentration between each candidate).

MAPKs play an important role in the regulation of inflammatory signaling cascades (Dong et al., 2020). We performed immunoblotting of phosphorylated forms of ERK1/2, p38, and JNK to analyze the upstream signaling of AT and ATF in response to TNF-α exposure. Cells were treated with AT or ATF at a dose range of 1–10 μg/mL. Then, TNF-α was incubated for the next 30 min to detect phosphorylated forms of p38 and ERK1/2. Phosphorylated JNK was observed after incubation with TNF-α for 5 h. TNF-α significantly increased the phosphorylation of ERK1/2, p38, and JNK, which were ameliorated by AT or ATF pretreatment (Figure 4).

**FIGURE 5:**
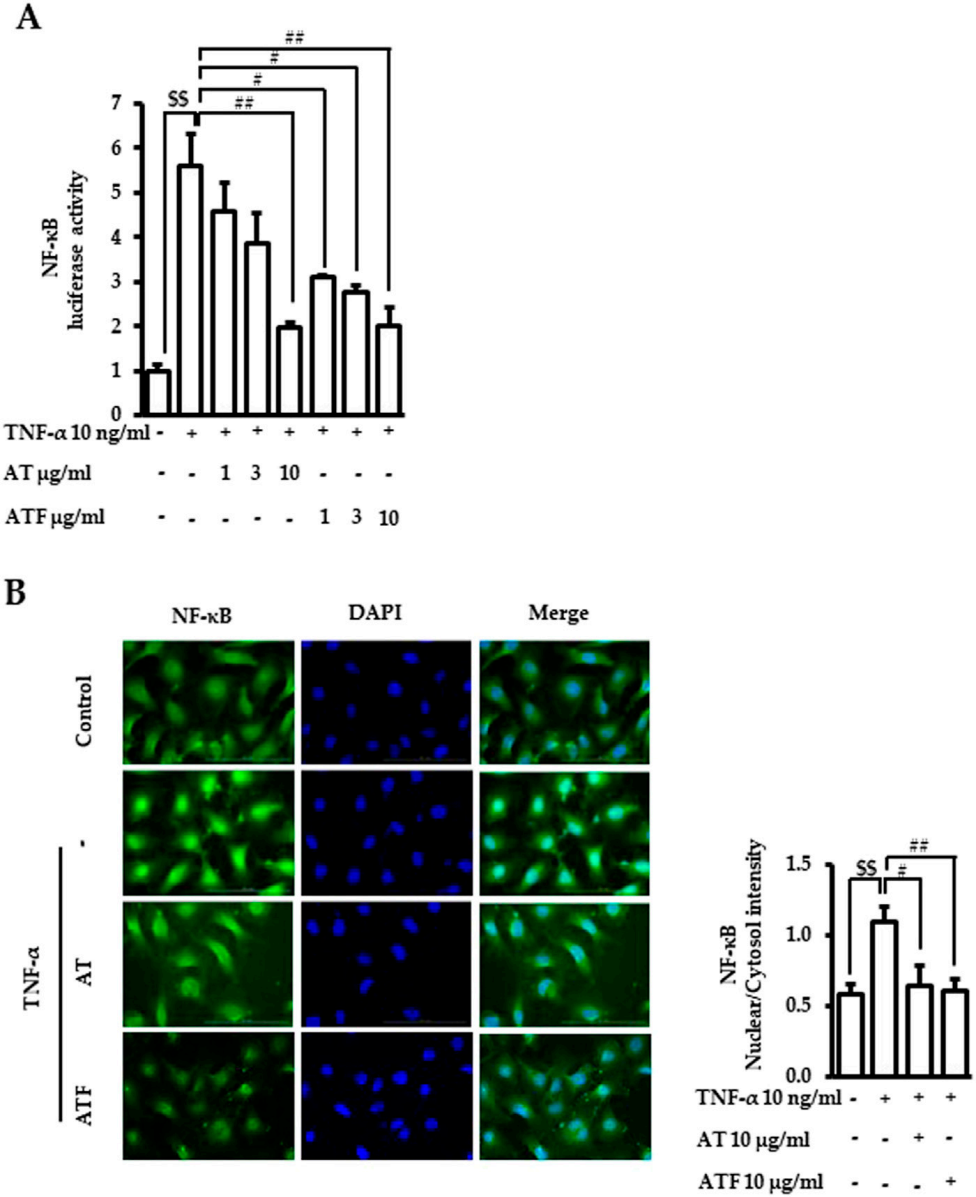
Inhibitory effects of AT and ATF on TNF-α-induced NF-κB transactivation and nuclear translocation. **(A)** Cells were transiently transfected with NF-κB promoter gene construct and subsequently treated with TNF-α for 18 h with or without AT or ATF pretreatment for 1 h. Luciferase activity was determined in lysates. The data were obtained from multiple analyses (n = 3). **(B)** Cells were pretreated with AT or ATF for 1 h and TNF-α for the next 1 h. Immunocytochemistry was carried out, as described in *Materials and methods*. NF-κB p65 was stained green, and nuclei were stained blue. The representative image (left panel) and the quantitative analyses of the fluorescence intensities (right panel) are shown. The data were obtained from multiple analyses (n = 5). (^$$^ *p* < 0.01 for control vs. TNF-α; ^#^ *p* < 0.05, ^##^ *p* < 0.01 for TNF-α vs. candidate-treated group).

NF-κB is a master transcriptional factor that regulates inflammatory gene expression (Baker et al., 2011). Therefore, the effects of AT and ATF on NF-κB transcriptional activation under TNF-α exposure were determined. Pretreatment of AT or ATF abrogated TNF-α-induced transcriptional activity of NF-κB with IC_50_ values of 3.77 μg/mL and 0.90 μg/mL, respectively (Figure 5A). TNF-α-induced translocation of NF-κB from the cytoplasm into the nucleus was observed, which was inhibited by AT or ATF (Figure 5B). These results suggest that AT and ATF inhibit the transcription of the inflammatory genes by blocking TNF-α-induced nuclear translocation and transcriptional activity of NF-κB.

**FIGURE 6:**
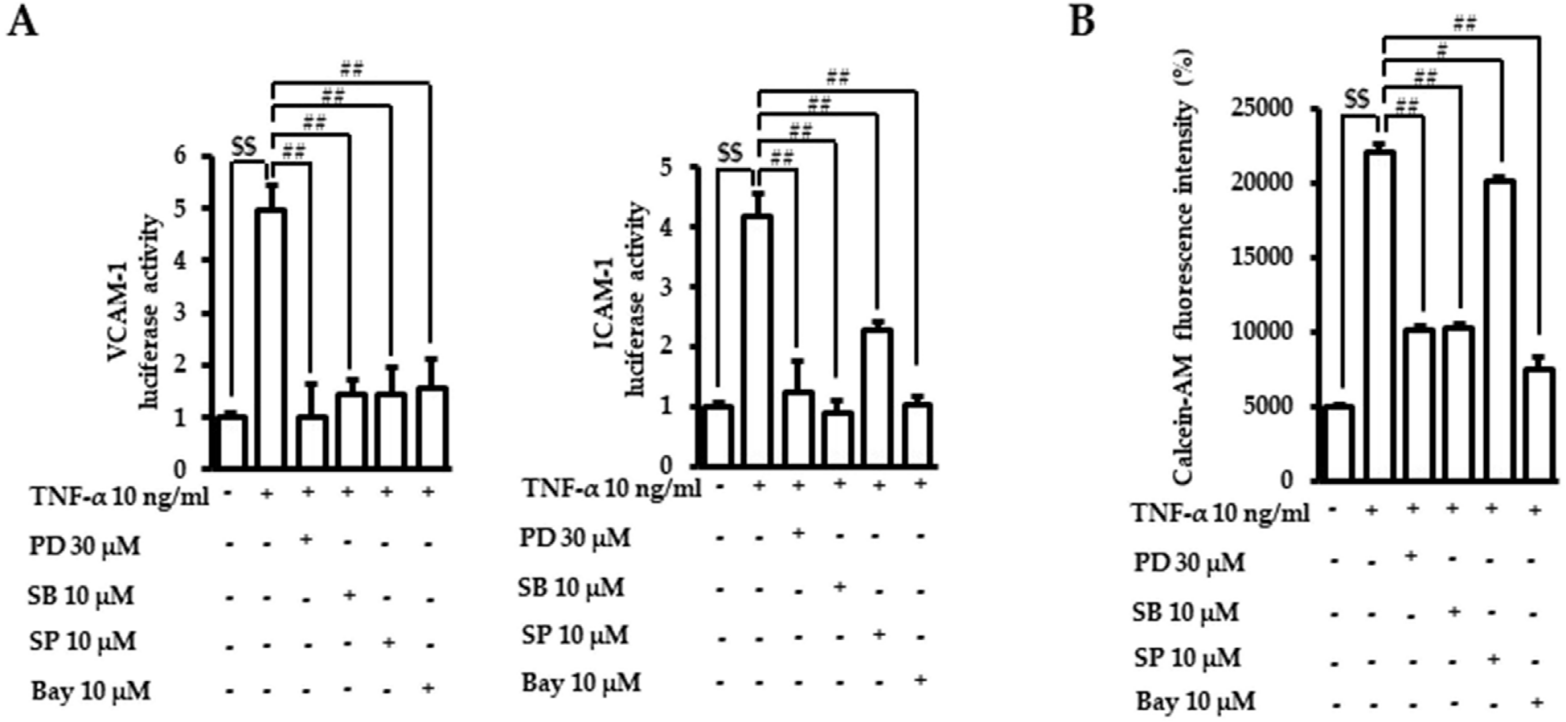
Role of MAPK/NF-κB in TNF-α-induced adhesion molecule transactivation and monocyte recruitment. **(A)** VCAM-1 or ICAM-1 promotor gene analyses were performed, as described in Figure 2B. Cells were treated with PD98059 (PD, ERK1/2 inhibitor), SB203580 (SB, p38 inhibitor), SP600125 (SP, JNK inhibitor), or Bay11-7082 (Bay, NF-κB inhibitor) for 1 h. Cells were then exposed to TNF-α for 18 h. The data were obtained from multiple analyses (n = 3). **(B)** Adhesion assays between monocytes and endothelial cells were performed, as described in Figure 1. After incubation with TNF-α with or without chemical inhibitors of MAPK and NF-κB, the intensity of the fluorescent calcein-AM signal was assessed with a fluorometer. The data were obtained from multiple analyses (n = 3). (^$$^ *p* < 0.01 for control vs. TNF-α; ^#^ *p* < 0.05, ^##^ *p* < 0.01 for TNF-α vs. candidate-treated group).

We then confirmed the role of MAPKs and NF-κB in the regulation of adhesion molecule expressions and adhesion between endothelial cells and monocytes using chemical inhibitors. Cells were preincubated with the indicated doses of inhibitors, including PD98059 (ERK1/2 inhibitor), SB203580 (p38 inhibitor), SP600125 (JNK inhibitor), and Bay11-7082 (NF-κB inhibitor), and following TNF-α incubation. The TNF-α-induced luciferase activities of adhesion molecules VCAM-1 and ICAM-1 were mitigated by inhibitors of MAPK and NF-κB (Figure 6A). The attachment of calcein-AM labeled monocytes to endothelial cells induced by TNF-α was also inhibited by the blocking of MAPKs and NF-κB (Figure 6B). These results confirmed that MAPK/NF-κB signaling pathways play an important role in TNF-α-regulated endothelial cell inflammation.

### 3.5 Inhibition of TNF-α-induced NF-κB acetylation by AT and ATF via the NF-κB and SIRT1 interaction

**FIGURE 7:**
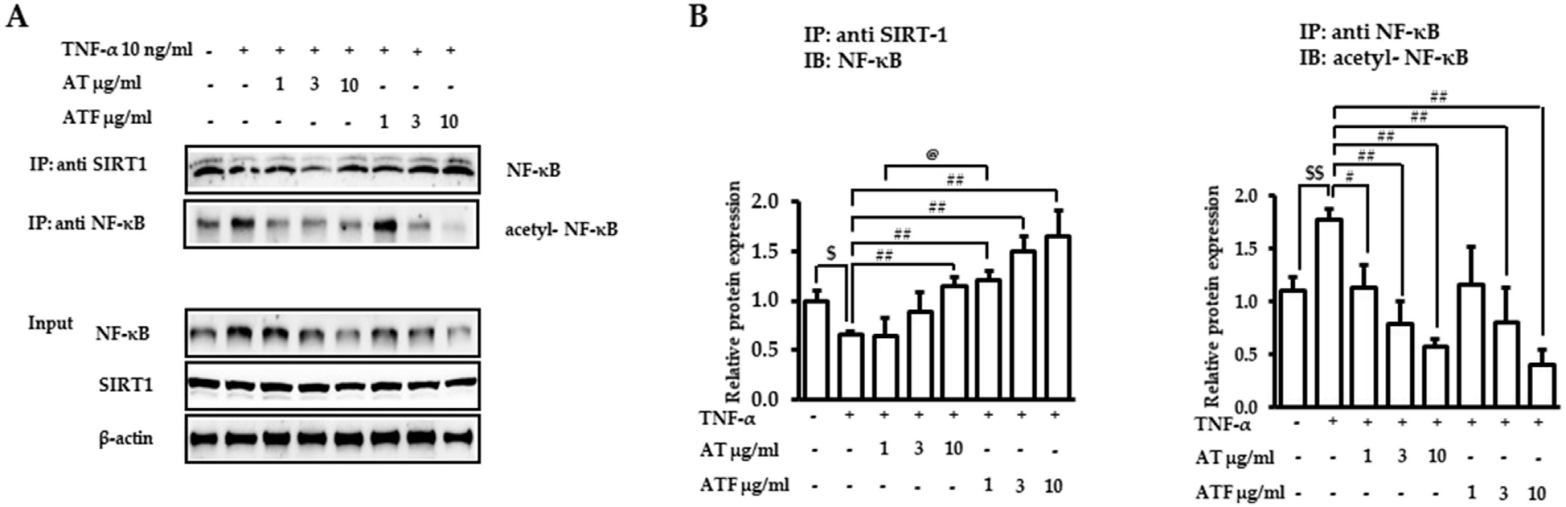
Interaction of NF-κB and SIRT1 induced by AT and ATF. **(A)** Cells were pretreated with AT or ATF for 1 h and then exposed to TNF-α for 30 min. The lysates were immunoprecipitated with an anti-SIRT1 antibody or anti-NF-κB antibody and then subjected to immunoblotting using anti-NF-κB and anti-p65 acetyl K310 antibodies, as described in the *Materials and methods* section. The NF-κB and SIRT1 levels in input samples were determined by immunoblotting. **(B)** Graphs represent relative densitometric intensities of NF-κB and acetylated NF-κB obtained from multiple analyses (n = 4). (^$^ *p* < 0.05, ^$$^ *p* < 0.01 for control vs. TNF-α; ^#^ *p* < 0.05, ^##^ *p* < 0.01 for TNF-α vs. candidate-treated group; ^@^ *p* < 0.05 for the same concentration between each candidate).

NF-κB posttranslational modification is a critical step in regulating inflammatory gene expression (Chaturvedi et al., 2011). SIRT1, an NAD^+^-dependent deacetylase, binds to NF-κB and inhibits transcriptional activity of NF-κB by deacetylating the RelA/p65 subunit at Lys^310^ (Yeung et al., 2004). Therefore, we tested whether SIRT1 was involved in AT or ATF-mediated NF-κB inhibition. NF-κB was bound to SIRT1 in the resting state, which failed to induce NF-κB transcriptional activity. The NF-κB/SIRT1 complex was dissociated in response to TNF-α, which was inhibited by AT or ATF with high potency in ATF (Figures 7A, B). TNF-α exposure resulted in the release of NF-κB from SIRT1 and increased acetylation of NF-κB at Lys^310^. AT or ATF inhibited the acetylation of Lys^310^ by promoting the interaction between NF-κB and SIRT1, thereby maintaining NF-κB in a deacetylated state. ATF significantly maintained the binding of NF-κB and SIRT1, implying that ATF exhibited a stronger effect in enhancing the NF-κB/SIRT1 interaction than AT did. Thus, the inhibitory effect of AT and ATF on NF-κB transactivation is due to the suppression of acetylated p65, which is attributed to SIRT1-mediated posttranslational modifications of NF-κB.

### 3.6 The role of SIRT1 in the protective effects of AT and ATF against TNF-*α* exposure

**FIGURE 8:**
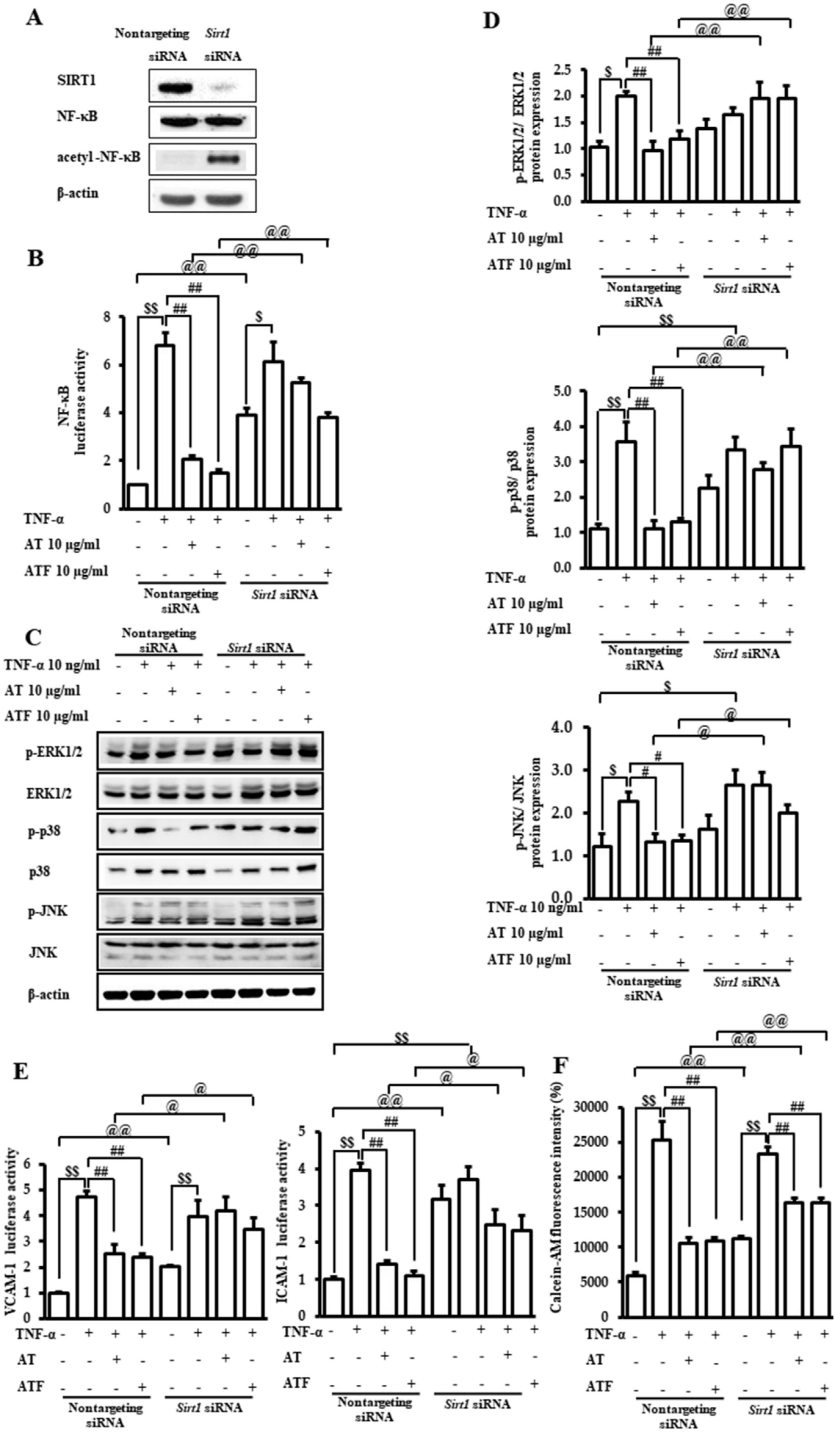
Role of SIRT1 in the protective effect of AT and ATF against TNF-α. Cells were transiently transfected with either non-targeting siRNA or *Sirt1* siRNAs. **(A)** Lysates were subjected to immunoblotting using anti-SIRT1, anti-NF-κB, and anti-p65 acetyl K310 antibodies. **(B)** Cells were pretreated with AT or ATF at the indicated concentrations for 1 h and then exposed to TNF-α for 18 h. NF-κB transactivation was analyzed with or without *Sirt1* gene knockdown using a reporter gene assay, as described in Figure 5A. Data were obtained from multiple analyses (n = 3). **(C)** Cells were incubated with AT or ATF for 1 h, followed by treatment of TNF-α for the indicated times, as shown in Figure 4. The representative expression levels of phosphorylated and total MAPKs in cell lysates were detected by Western blotting. **(D)** Graphs representing the relative densitometric ratio of each p-ERK, p-p38 MAPK, and p-JNK were normalized to their respective total EKR, p38 MAPK, and JNK levels (n = 4). Data were obtained from multiple analyses (n = 4). **(E)** VCAM-1 and ICAM-1 luciferase activities were measured, as described in Figure 2B. Data were obtained from multiple analyses (n = 3). **(F)** Attachment of monocytes to endothelial cells was evaluated based on fluorescence intensities, as shown in Figure 1. Data were obtained from multiple analyses (n = 3). (^$^ *p* < 0.05, ^$$^ *p* < 0.01 for control vs. TNF-α; ^#^ *p* < 0.05, ^##^ *p* < 0.01 for TNF-α vs. candidate-treated group; ^@^ *p* < 0.05, ^@@^ *p* < 0.01 for each candidate between non-targeting siRNA group and *Sirt1* siRNA group).

The role of SIRT1 in the AT and ATF against TNF-α-mediated MAPK/NF-κB signaling pathway and the regulation of adhesive molecules were evaluated by *Sirt1* gene knockdown. Silencing *Sirt1* significantly reduced its expression (Figure 8A). SIRT1-deficient cells increased NF-κB transactivation along with K310 acetylation of RelA/p65 in the absence of TNF-α, which is consistent with previous findings (Nguyen et al., 2019). The inhibitory effects of AT and ATF on NF-κB transactivation in response to TNF-α were diminished in SIRT1-deficient cells compared to the control group, indicating that SIRT1 is required for AT and ATF to suppress NF-κB acetylation (Figure 8B). Additionally, the differential regulation of MAPK phosphorylation by AT and ATF was attenuated in *Sirt1* siRNA-transfected cells (Figures 8C, D). In these cells, AT and ATF also failed to inhibit TNF-α-induced VCAM-1 and ICAM-1 transactivations (Figure 8E). Furthermore, *Sirt1* silencing mitigated the inhibitory effects of AT and ATF on TNF-α-induced adhesion, as measured by the fluorescent calcein-AM signal on endothelial cells, which is consistent with NF-κB regulation (Figure 8F). These findings suggest that SIRT1 plays a critical role in the protective effects of AT and ATF against TNF-α-mediated vascular inflammation.

### 3.7 SIRT1 targeting of AT, ATF, and their bioactive compounds

**FIGURE 9:**
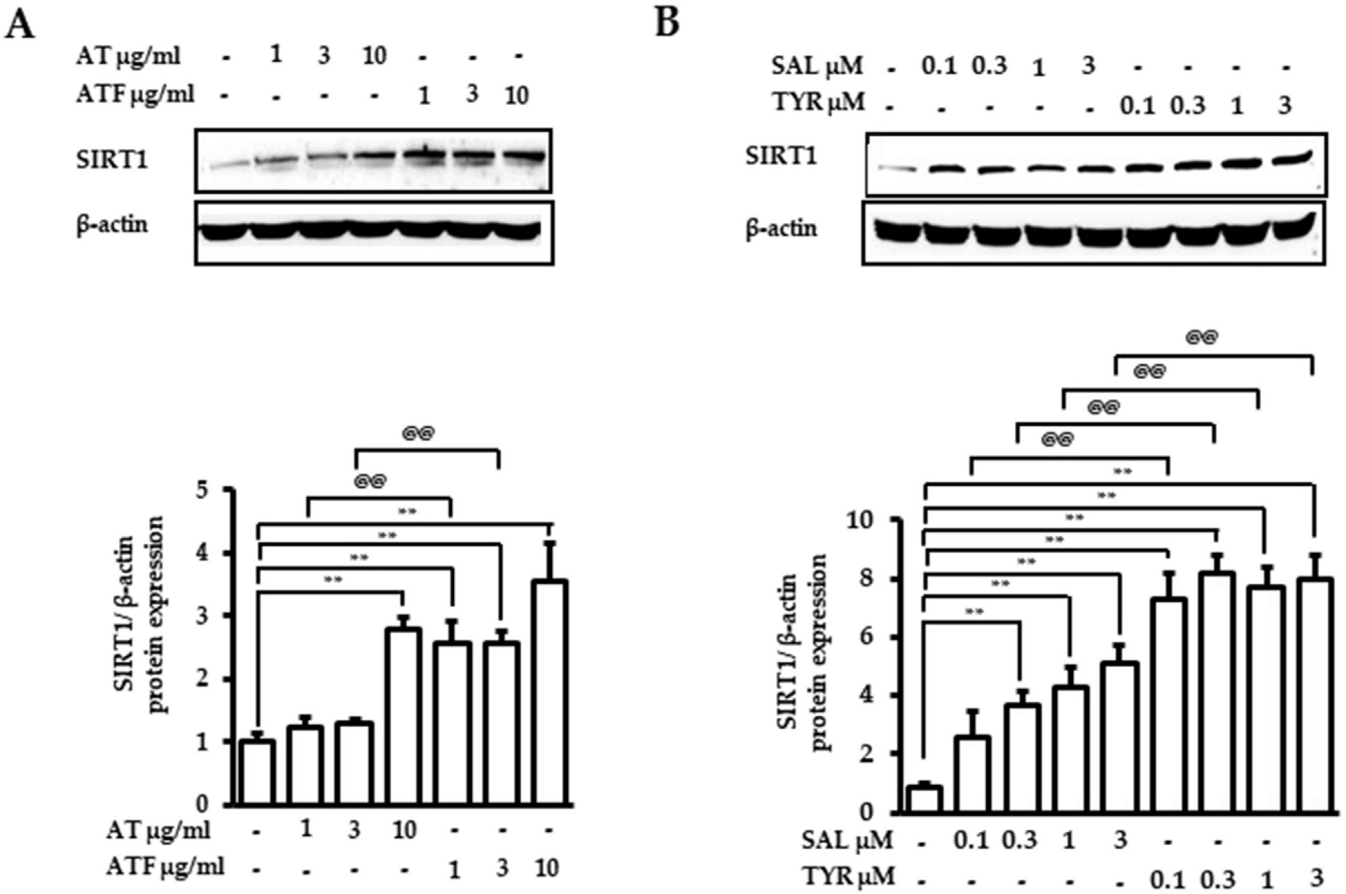
Effects of AT, ATF, SAL, and TYR on the SIRT1 expression. Cells were treated with **(A)** AT, ATF, **(B)** SAL, and TYR at the indicated concentration for 24 h. Cells lysates were used for Western blot. The representative blots of SIRT1 are shown in the upper panel, and graphs indicate relative densitometric intensities obtained from multiple analyses (n = 4). (^**^ *p* < 0.01 for control vs. candidate-treated group; ^@@^ *p* < 0.01, for the same concentration between each candidate).

We found that SIRT1 is an important target of AT or ATF for regulating the anti-inflammatory responses. We next determined SIRT1 expression by AT, ATF, SAL, or TYR after 24 h. Compared to AT, ATF significantly increased the expression of SIRT1 by 3.5-fold (Figure 9A). SAL and TYR also exhibited 5-fold and 8-fold upregulation of SIRT1 protein expression, respectively, supporting the idea that these candidates increase both SIRT1 activity and SIRT1 expression (Figure 9B).

**FIGURE 10:**
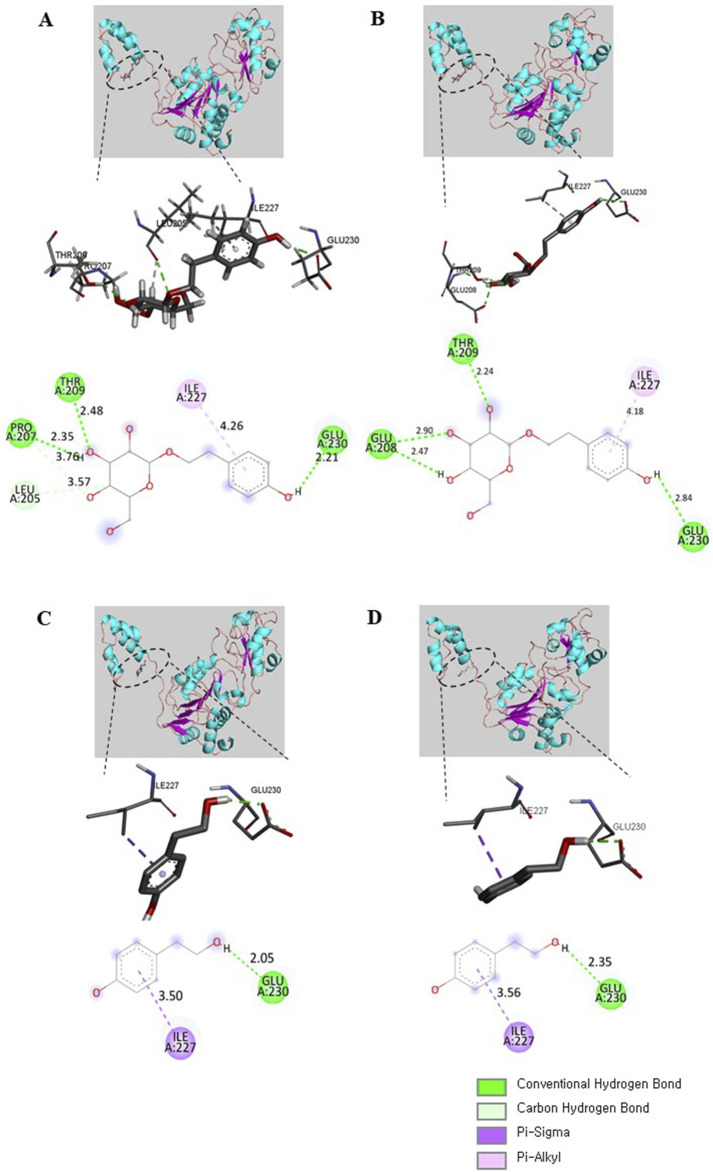
SAL and TYR binding to SIRT1 NTD. The binding modes of SAL with **(A)** 4ZZH and **(B)** 4ZZJ or TYR with **(C)** 4ZZH and **(D)** 4ZZJ are illustrated. The interactions of SAL or TYR on the crystal structures are displayed in 3D and 2D diagrams. Deep green, conventional hydrogen bond; light green, carbon hydrogen bond; deep purple, pi–sigma bond; light purple, pi–alkyl bond.

We next performed molecular docking to analyze how SAL or TYR (the active compounds in AT and ATF) interacts with SIRT1. The multiple alignment results of hSIRT1 and two SIRT1 homologous residues (PDB IDs 4ZZH and 4ZZJ) indicate that all structures show strong homology (>90%) with the NTD and the catalytic domains of hSIRT1 (Supplementary Figure 3). The interaction of bioactive compounds with the allosteric region of the NTD can initiate conformational changes, leading to stabilization and induction of deacetylase activity of SIRT1 (Bonkowski and Sinclair, 2016). Hence, we generated molecular docking of the NTD region of SIRT1 with SAL or TYR, and detailed interactions were illustrated; all four docking results showed the same binding pocket regions (Figure 10). The glycol moiety of SAL interacted with SIRT1 via hydrogen bonds to Leu^205^, Pro^207^, Thr^209^ (4ZZH), Glu^208^, and Thr^209^ (4ZZJ); tyrosol residue of SAL showed interactions with Ile^227^ and Glu^230^ (Figures 10A, B). Interestingly, TYR also interacted with 4ZZH and 4ZZJ at Ile^227^ and Glu^230^, which were identical to the tyrosol residue of SAL (Figures 10C, D). These docking data indicate that the tyrosol moiety in both SAL and TYR bind to the same binding pocket of SIRT1 and that the binding distance toward SIRT1 is closer in TYR than in SAL, which could potentially regulate SIRT1 activity.

### 3.8 Molecular dynamics (MD) analysis of the docked SIRT1–SAL and SIRT1–TYR models

**FIGURE 11:**
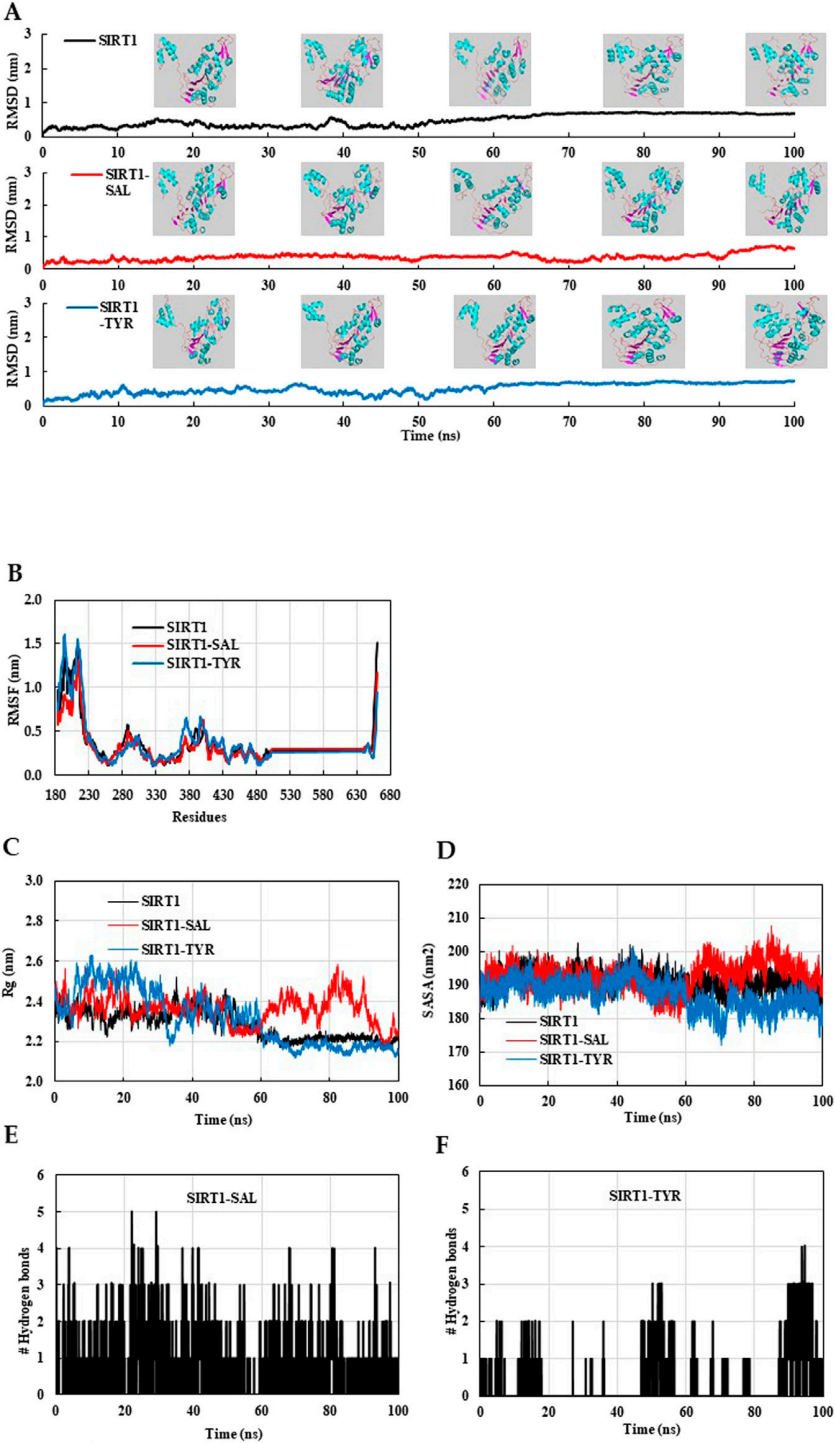
MD simulation analysis of native SIRT1, SIRT1–SAL and SIRT1–TYR models. The final MD trajectories of native SIRT1, SAL bound SIRT1, and TYR bound SIRT1 were analyzed over a 100-ns simulation. **(A)** RMSD plot of the SIRT1 in complex with SAL and TYR. **(B)** Residual fluctuation (RMSF) plot of SIRT1 before and after SAL and TYR binding. **(C)** Time evolution of the radius of gyration (Rg), **(D)** solvent-accessible surface area (SASA) plot, **(E)** Hydrogen bonds of the SAL–SIRT1 complex, and **(F)** Hydrogen bonds of the TYR–SIRT1 complex.

We evaluated the structural stability and dynamic behavior of ligand–protein complexes using MD simulation. The RMSD plot showed that the initial fluctuations of native SIRT1 and SIRT1–TYR persisted up to 50 ns, and then, the structures stabilized up to 100 ns (Figure 11A). Interestingly, the SIRT1–TYR complex showed movement of the allosteric region toward the catalytic region of SIRT1 after 80 ns, suggesting that TYR’s interaction with SIRT1 NTD may contribute to SIRT1 activation (Figure 11A, inset). The average RMSD and RMSF of SIRT1–SAL were 0.373 nm and 0.343 nm, respectively, which were lower than those of SIRT1–TYR (Table 1). The initial fluctuations in the RMSF plot of combined trajectories may be due to the random interactions in the NTD region. TYR interacted with the SIRT1 NTD, vibrating more strongly than SAL. The slight vibration of the TYR–SIRT1 complex in the catalytic region indicates the role of TYR in the regulation of SIRT1 interaction (Figure 11B). Next, the trajectories of the MD simulation were analyzed by Rg, SASA, and the number of hydrogen bonds. The Rg plot revealed distinct deviation patterns between the SAL–SIRT1 and TYR–SIRT1 complexes (Figure 11C). The Rg values of SIRT1–SAL and SIRT1–TYR were unstable during the initial 40 ns. After 60 ns, the Rg of SIRT1–TYR stabilized and remained below 2.2 nm, which was lower than the Rg of SIRT1–SAL (2.2–2.6 nm). The mean SASA value of SIRT1–TYR was the lowest compared to native SIRT1 and SIRT1–SAL (Table 1). The SASA plot showed that SIRT1–SAL fluctuated after 60 ns, suggesting slight flexibility of SIRT1 upon ligand binding. In contrast, the SASA value of SIRT1–TYR significantly decreased compared to that of SIRT1–SAL at the end of the simulation (after 60 ns) (Figure 11D). SAL exhibited a higher number of hydrogen bonds with SIRT1, with a maximum of five bonds, while TYR showed a more variable number of hydrogen bonds throughout the simulation (Figures 11E, F). These MD results revealed important insights into the interaction of SAL and TYR with SIRT1, suggesting that SAL and TYR have different interaction mechanisms with SIRT1.

**TABLE 1:** MD parameters for native SIRT1, SAL–SIRT1, and TYR–SIRT1 systems.

Complex	Average RMSD (nm)	Average RMSF (nm)	Average Rg (nm)	Average SASA (nm^2^)
4ZZJ	0.482	0.390	2.285	191.176
SAL–4ZZJ	0.373	0.343	2.364	192.455
TYR–4ZZJ	0.510	0.400	2.315	187.198

### 3.9 Effects of AT and ATF on the STZ-treated mouse retina

**FIGURE 12:**
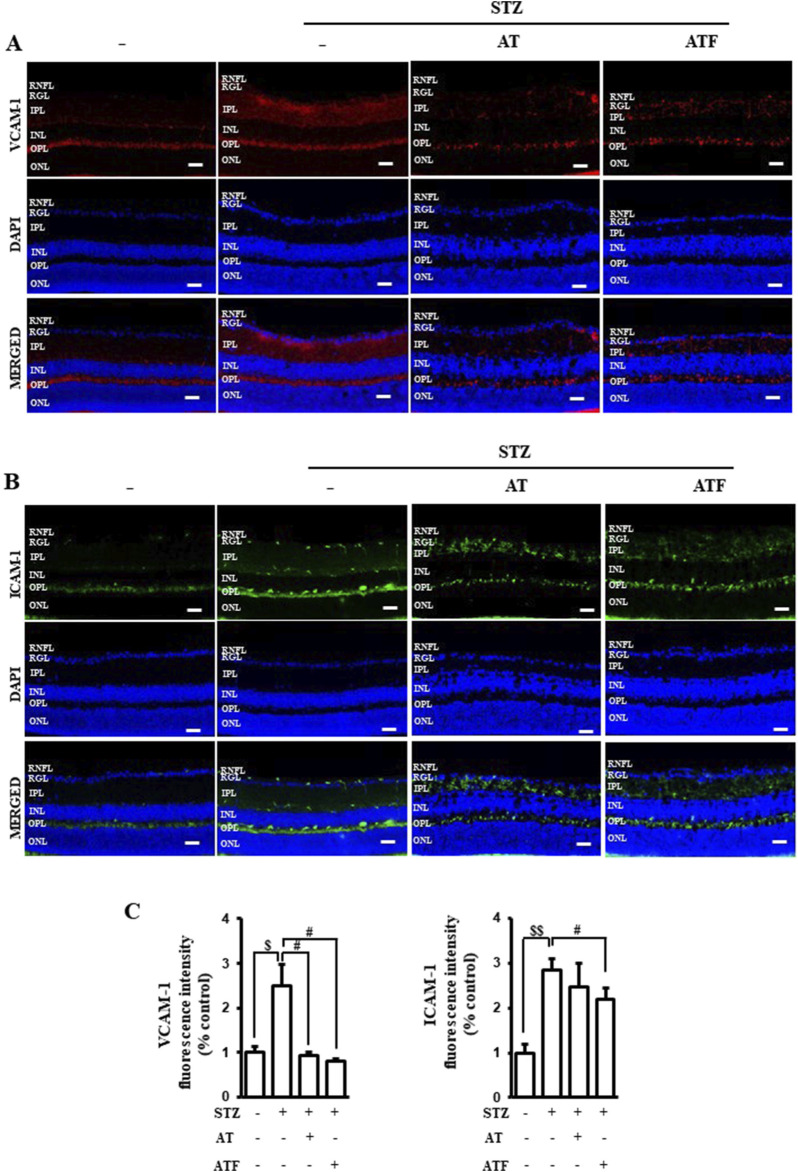
Effects of AT and ATF on STZ-treated retinal adhesion molecules. Representative immunostainings of **(A)** VCAM-1 and **(B)** ICAM-1 in retinal sections from STZ-induced or STZ + AT and STZ + ATF (50 mg/kg)-treated groups are shown. **(C)** Quantifications of VCAM-1 and ICAM-1 positive intensity in the retina are shown. Nuclei were identified using 4′,6-diamidino-2-phenylindole (DAPI) (blue). STZ, streptozotocin; RNFL, retinal nerve fiber layer; RGL, retinal ganglion cell layer; IPL, inner plexiform layer; INL, inner nuclear layer; OPL, outer plexiform layer; ONL, outer nuclear layer. Scale bar 100 μm.

Diabetic retinopathy (DR) is characterized by retinal nerve deformation and microcirculatory disturbances caused by an inflammatory process (Gerhardinger et al., 2005; Yang et al., 2022). Hyperglycemia induces adhesive molecules like VCAM-1 and ICAM-1, which contribute to damage in the retina, the neuro-vascular coupling tissue. Therefore, we evaluated the levels of VCAM-1 and ICAM-1 in STZ-treated retinal sections. STZ induced VCAM-1 in the inner plexiform layer of the retina (Figures 12A, C). AT and ATF attenuated STZ-induced VCAM-1 expression. ICAM-1 expression increased in the retinal nerve fiber layer of the STZ-treated retina sections, and AT and ATF moderately inhibited ICAM-1 distribution in this layer, with ATF showing a more potent effect (Figures 12B, C). These results suggest that AT and ATF reduce STZ-induced diabetic retina damage by inhibiting adhesion molecules.

## 4 Discussion

AT is a medicinal herb used in Asian countries to treat various diseases (Yu et al., 2010; Park et al., 2019; Cho et al., 2020; Bae et al., 2022). AT exhibits antioxidant, anti-inflammatory, and anti-lipogenic properties and outstanding protective effects against liver diseases (Yu et al., 2010; Park et al., 2019). Recently, we reported that AT significantly blocked cholestasis-induced liver damage via the inhibition of inflammation and fibrosis (Bae et al., 2022). In hepatic diseases such as hepatitis and cirrhosis, elevated systemic and hepatic TNF-α levels have been linked to vascular dysfunction (Meng et al., 2022). Endothelial cells in a vessel are a major component cell type involved in vascular function since they act as the first barrier against injury (Kitada et al., 2016). Endothelial dysfunction is involved in vasoinflammation, leading to complications such as retinal microvascular damage (Lenin et al., 2018; Ouyang et al., 2024). No studies have been reported on whether AT exerts an inhibitory effect on vascular inflammation. The present study is the first to assess the protective effect of AT against TNF-α-induced endothelial inflammation.

Inflammatory processes, including monocyte adhesion to endothelial cells, are considered early key events and critical steps in response to stimuli (e.g., TNF-α) (Nguyen et al., 2019). AT suppressed TNF-α-induced monocyte adhesion to endothelial cells at the IC_50_ value of 1.62 μg/mL. SAL, the main compound of AT, also suppressed adhesion with the IC_50_ value of 0.94 μM, indicating that SAL acts as a bioactive compound in AT. Surprisingly, TYR showed higher potency than SAL against TNF-α-induced adhesion (IC_50_ = 0.14 μM). SAL constitutes 6%–8% of the extracts obtained from various parts of the AT plant (Hwang et al., 2013; Park et al., 2019). The TYR content in AT is much less compared to SAL (Hwang et al., 2013). Based on the IC_50_ (potency) differences between SAL and TYR, we considered the possibility that the antiadhesive effect of AT could be improved by converting SAL of AT to TYR. Fermented soybeans with *B. subtilis* resulted in the highest concentration of isoflavone aglycones and, thus, a decrease in isoflavone glucosides (Huynh et al., 2014). *Bacillus subtilis* shows potential to release phenolic acids and flavonoids from plant sources, enhancing the yield of polyphenols. Additionally, it can produce β-glucosidase, which facilitates the deglycosylation of SAL. Therefore, we carried out AT fermentation using *B. subtilis*. AT fermentation resulted in an increase in TYR and a decrease in SAL. The adhesion assay indicated that ATF with an IC_50_ value of 0.49 μg/mL is better in inhibiting monocyte adhesion to endothelial cells compared to AT with an IC_50_ value of 1.62 μg/mL. These findings suggest that ATF may be a promising protective product against vasoinflammation.

Most herbal medicinal plants containing polyphenols are administered orally. Plant phenols often exist as glycosides, which have the potential of deglycosylation, producing its aglycone metabolites in the gastrointestinal system *in vivo*. The content of SAL is the active standard component to evaluate the quality of AT (Hwang et al., 2013). In the pharmacokinetic study, oral administration of SAL *in vivo* failed to show deglycosylation of SAL into TYR, whereas TYR was identified after intravenous injection of SAL (Guo et al., 2012). The aglycone metabolite, TYR, has potent pharmacological properties, but it is difficult to be biotransformed from SAL of AT in the body. These findings are consistent with previous reports showing that only 2% SAL exists in plasma as the aglycone metabolite TYR (Guo et al., 2012). In this current study, the fermentation processes with *B. subtilis* resulted in microbial conversion. We focused on the conversion of SAL (the main compound in AT) to TYR, although various metabolites can be produced by microbial fermentation. The stability of SAL and TYR is stable without degradation under all storage conditions, suggesting that those substances are resistant to breakdown in the fermented solution (Guo et al., 2012).

Vascular inflammation could be initiated when monocytes circulating in the bloodstream are recruited to sites of inflammation in the vascular endothelium (Nguyen et al., 2019). AT and ATF significantly suppressed TNF-α-induced transactivation and expression of VCAM-1 and ICAM-1 in human endothelial cells. The production of MCP-1, a proinflammatory chemokine, was also significantly inhibited by AT and ATF. Chemokines are secreted in response to signals such as pro-inflammatory cytokines (including TNF-α), where they play an important role in selectively recruiting monocytes (Nguyen et al., 2019). These results suggest that vascular endothelial cells are a major action site of AT and ATF to prevent the early stages of vascular inflammation. ATF is more effective in blocking TNF-α-induced adhesion molecules and MCP-1 in endothelial cells. This enhanced anti-inflammatory effect of ATF seems to be due to the pharmacological potency of TYR and its longer T_1/2_ than SAL (Guo et al., 2012).

Multiple MAPK signaling cascades operate in a large modular network that regulates inflammation (Schulze-Osthoff et al., 1997). MAPKs, including ERK, p38 MAPK, and JNK, are phosphorylated by cytokines including TNF-α, which is associated with inflammatory gene expression. We found that TNF-α increased phosphorylated ERK and p38 MAPK at early time points and JNK phosphorylation at a late time point in endothelial cells, which is consistent with a previous report (Lo et al., 2014). AT and ATF significantly inhibited TNF-α-mediated ERK and p38 MAPK phosphorylation. However, AT and ATF showed a weak inhibitory effect on TNF-α-mediated JNK activation, suggesting that these candidates differentially regulate the MAPK pathway. AT itself increased MAPK phosphorylation in hepatocytes, whereas AT significantly suppresses ERK and p38 MAPK in endothelial cells, demonstrating the controversy of AT in the regulation of the MAPK pathway (Kim et al., 2015; Zou et al., 2015; Park et al., 2019). These results claim that AT may differentially affect MAPKs depending on the cell type and organ.

NF-κB is a key transcriptional factor that regulates inflammatory genes (Liu et al., 2017). The NF-κB motif in the promoters of ICAM-1, VCAM-1, and MCP-1 is particularly important for the induction of these genes (Rothgiesser et al., 2010; Meng et al., 2022). AT and ATF inhibited the TNF-α-induced nuclear translocation and transcriptional activity of NF-κB. The transcriptional activity of NF-κB is regulated by RelA/p65 posttranslational modifications such as phosphorylation, methylation, ubiquitination, and acetylation (Yang et al., 2010). Among these, the acetylation at Lys^310^ of RelA/p65 is crucial because it is required for the full transcriptional activity of NF-κB (Chen et al., 2002). SIRT1, a deacetylase enzyme, selectively interacts with RelA/p65 to mediate the deacetylation of Lys310 in RelA/p65, thus inhibiting NF-κB transcriptional activity (Yeung et al., 2004). We previously reported that TNF-α-stimulated NF-κB activation was regulated by the interaction between NF-κB and SIRT1 (Nguyen et al., 2019). In this study, AT and ATF reduced the TNF-α-induced dissociation of the NF-κB/SIRT1 complex, resulting in deacetylation of RelA/p65 and a subsequent decrease in NF-κB transcriptional activity. ATF showed stronger effect at lower doses compared to AT. These results suggest that SIRT1, a transcriptional regulator of NF-κB, may serve as a direct target of AT and ATF. Additionally, we confirmed the role of SIRT1 in the inhibition of NF-κB and inflammatory responses by AT and ATF using the *SIRT1* gene knockdown experiment.

Overexpression of SIRT1 and treatment with STAC improves the physiological function of SIRT1 (Bonkowski and Sinclair, 2016; Chen et al., 2024). AT and ATF significantly upregulated SIRT1 expression, with higher levels in ATF. TYR significantly increased SIRT1 expression, with higher potency and efficacy than SAL. The enhanced increase of SIRT1 expression by ATF and TYR supports the idea that the tyrosol moiety may be important for increasing the activity of SIRT1 and stabilizing its deacetylase activity.

The proven protective role of increased SIRT1 activity in various dysfunctions makes this enzyme a promising therapeutic target (Dai et al., 2015). A single point mutation of the Glu^230^ residue of NTD in SIRT1 has been shown to impair activation by direct allosteric activation of SIRT1 or tight substrate binding of SIRT1 and substrate by STAC, demonstrating the importance of the NTD and the Glu^230^ residue for SIRT1 activation by STAC (Dai et al., 2015; Hou et al., 2016; Liu et al., 2023). The SIRT1 NTD can physically modulate the association of SIRT1 and NF-κB, thereby decreasing the acetylation of NF-κB and suppressing inflammation (Ghisays et al., 2015). Medical plants selected as the SIRT1 activator form hydrophobic interactions with Ile^223^ and Ile^227^, while nicotinamide (as a SIRT1 inhibitor) fails to interact with Ile^227^, suggesting that Ile^223^ and Ile^227^ could be key residues (Azminah et al., 2019). In molecular docking, the hydroxyl groups of SAL or TYR form hydrogen bonds with the side chain of the Glu^230^ residue in NTD of SIRT1. The distances of TYR toward Ile^227^ and Glu^230^ residues of SIRT1 were closer than those observed for the tyrosol moiety of SAL, suggesting that TYR might have a higher binding affinity for the SIRT1 NTD. MD simulations showed that SAL forms strong interactions with the NTD of SIRT1, stabilized by multiple hydrogen bonds. Interestingly, while TYR forms fewer hydrogen bonds with SIRT1, its secondary structure remains more compact, indicating a potential resistance to unfolding in solvent. The sustained stabilities of the SAL-bound and TYR-bound SIRT1 complexes indicate that both SAL and TYR interact favorably with the allosteric region of SIRT1, inducing conformational changes that enhance its deacetylase activity. The SIRT1–TYR complex exhibited a shift in the allosteric region toward the catalytic region of SIRT1 and displayed high fluctuation in the catalytic region of SIRT1. These results suggest that the interaction with TYR increases the flexibility of this region, leading to conformational changes that promote SIRT1 activity.

Notably, the association of NF-κB/SIRT1 and the deacetylation of p65 by ATF were observed to be higher than AT. This study suggests that TYR may be more potent in regulating SIRT1 activity, based on both structural and biochemical evidences. Further investigation is required to assess whether TYR activates SIRT1 toward the substrate RelA/p65, which may be crucial for the tyrosol moiety-dependent stimulation of RelA/p65 deacetylation. We plan to explore this in our upcoming research.

Diabetic retinopathy is a vascular disorder of the retina (Gerhardinger et al., 2005). STZ-induced hyperglycemia triggers the activation of the vascular endothelial layer of the inner blood–retinal barrier, leading to impairment of retina, a neurovascular coupling tissue (Lenin et al., 2018). In the present study, AT and ATF suppressed STZ-induced retinal adhesion molecules, consistent with the findings of cell studies. These results suggest a potential protective role on the increased leukocyte adhesion and transmigration across the blood–retinal barrier into the retinal tissue of these candidates against diabetic retinopathy. Expanding this research to clinical trials focused on diabetic retinopathy would be an important next step to confirm the validity of these finding.

## 5 Conclusion

**FIGURE 13:**
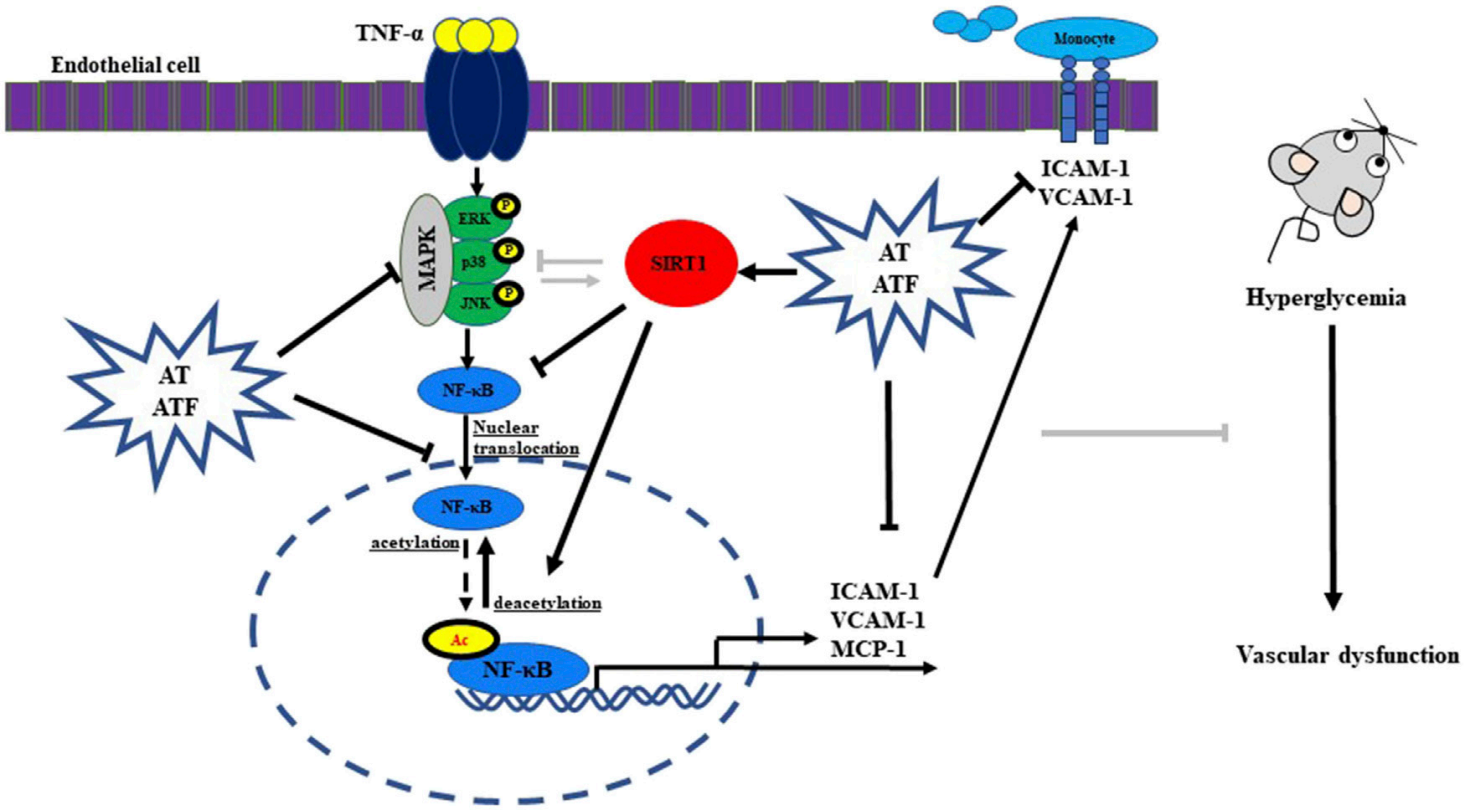
Schematic summary of the protective effects of AT and ATF.

We demonstrated that AT and ATF exert a protective effect against TNF-α-induced endothelial dysfunction. AT and its compounds, salidroside and tyrosol, attenuate TNF-α-mediated monocyte-endothelial cell adhesion and inhibit key biomarkers of endothelial inflammation, including adhesion molecules (VCAM-1 and ICAM-1) and the pro-inflammatory cytokine MCP-1 and NF-κB transactivation via an interaction with SIRT1. Notably, fermentation of AT with *B. subtilis* converts salidroside into tyrosol, which exhibits greater potential in suppressing endothelial inflammation (Figure 13). These findings have important implications for fermentation strategies aimed at producing high-value extracts from plants, such as ATF, with potential effects in inhibiting vascular inflammation. Additionally, our results reveal that the tyrosol moiety of salidroside and tyrosol targets the Glu^230^ residue of SIRT1 NTD, which inhibits NF-κB acetylation. These insights support the rational design of new candidates targeting the Glu^230^ residue in SIRT1 NTD to inhibit endothelial inflammation and related complications in metabolic syndrome.

## Data Availability

The raw data supporting the conclusions of this article will be made available by the authors, without undue reservation.

## Supplementary material

SUPPLEMENTARY FIGURE 1 Cell viabilities of **(A)** AT, ATF, **(B)** SAL, and TYR. Cell viability was measured by MTT assay. The data obtained from multiple analyses (n = 3).

SUPPLEMENTARY FIGURE 2 Representative HPLC chromatograms of AT and ATF. AT, ATF samples, and standard controls (SAL and TYR) were separated using HPLC. Column: C18 (4.6 mm × 250 mm, 5 µm particle size); temperature: 25°C; mobile phase: 0.1% formic acid in H_2_O **(A)** and MeOH 100%, flow rate: 1.0 mL/min; detection: λ 225/ 278 nm.

SUPPLEMENTARY FIGURE 3 Homology modeling of SIRT1. **(A)** Schematic diagram showing functional domains in full-length hSirt1. **(B)** Multiple sequence alignment of the hSIRT1 sequence and PDB IDs 4ZZH and 4ZZJ. NTD, N-terminal domain; CTD, C-terminal domain.

## Abbreviations

AT, *Acer tegmentosum* Maxim; ATF, AT fermented product; ICAM-1, intercellular adhesion molecule-1; MD, molecular dynamics; NTD, N-terminal domain; Rg, radius of gyration; RMSD, root-mean-square deviation; RMSF, root-mean-square fluctuation; SAL, salidroside; SASA, solvent-accessible surface area; SDS-PAGE, sodium dodecyl sulfate–polyacrylamide gel electrophoresis; SIRT1, sirtuin-1; STAC, sirtuin-activating compounds; STZ, streptozotocin; TNF, tumor necrosis factor; TYR, tyrosol; VCAM-1, vascular cell adhesion molecule-1.
