# Supplementary figures and images for: Acer tegmentosum Maxim and Bacillus subtilis-fermented products inhibit TNF-α-induced endothelial inflammation and vascular dysfunction of the retina: the role of tyrosol moiety in active compounds targeting Glu230 in SIRT1
Source: Front Pharmacol. 2024 Nov 20;15:1392179. doi: 10.3389/fphar.2024.1392179 (PMC11614635; doi:10.3389/fphar.2024.1392179)

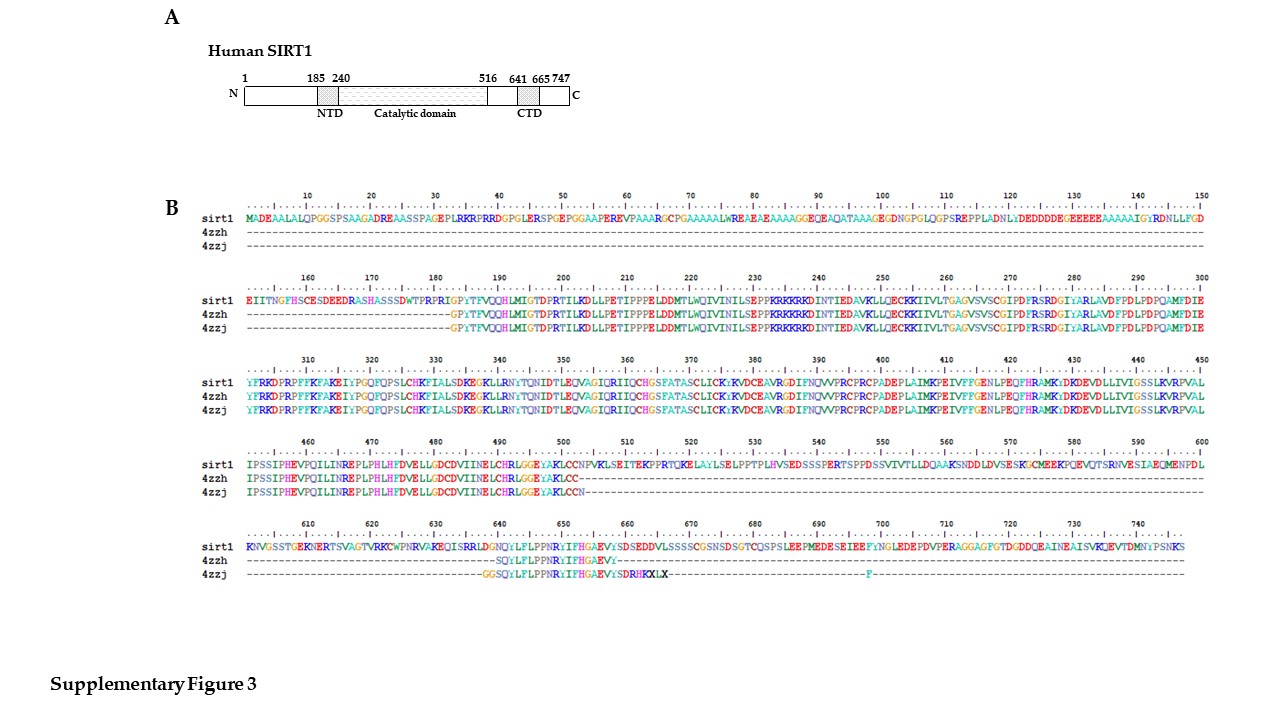

Supplement: Supplementary file 1 [file Image3.JPEG]

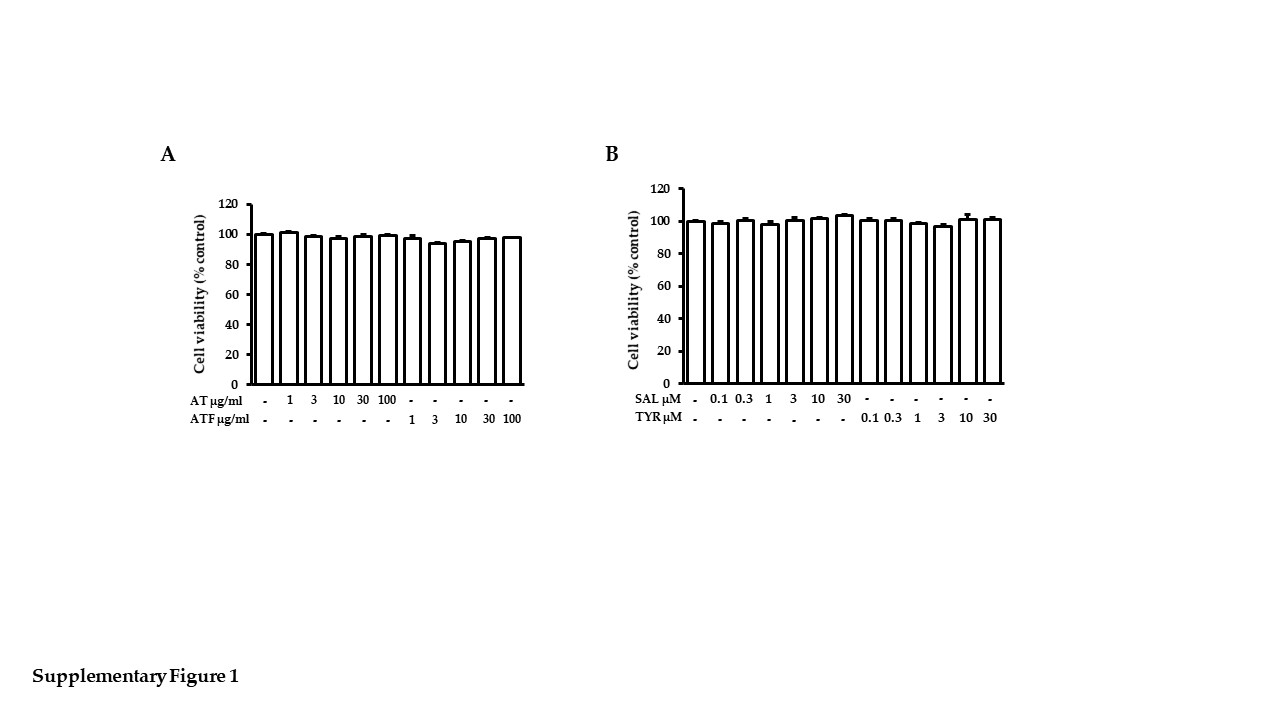

Supplement: Supplementary file 2 [file Image1.JPEG]

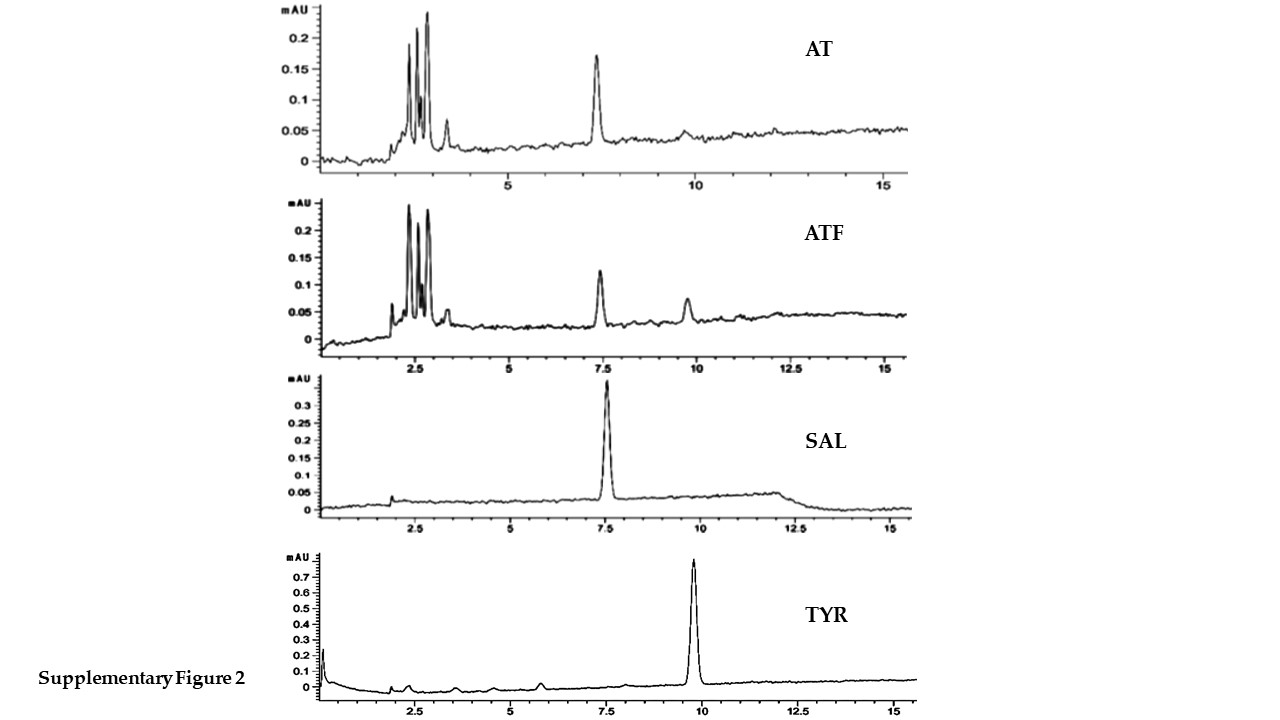

Supplement: Supplementary file 3 [file Image2.JPEG]
